# Topological Structure of Epigenetic Forests in Flower Morphogenesis

**DOI:** 10.1007/s11538-026-01688-2

**Published:** 2026-06-24

**Authors:** Yuriria Cortés-Poza, J. Rogelio Pérez-Buendía

**Affiliations:** 1 IIMAS, Unidad Académica de Yucatán, Universidad Nacional Autónoma de México (UNAM), Yuc, México; 2 SECIHTI – Centro de Investigación en Matemáticas (CIMAT), Unidad Mérida, Yuc, México

**Keywords:** Gene regulatory networks, Flower morphogenesis, Discrete dynamical systems, Epigenetic landscapes, Mathematical biology, Boolean networks, Attractor basins, Primary: 92B05, 37N25. Secondary: 92C42, 05C20, 94A17

## Abstract

Understanding how stable developmental patterns emerge from gene regulatory networks remains a central problem in developmental biology. Here, we study how classical homeotic mutations reshape the epigenetic landscape of the floral gene regulatory network of *Arabidopsis thaliana*. We represent this landscape as an Epigenetic Forest: a collection of rooted in-arborescences induced by the state transition graph of a Boolean gene regulatory network, where each tree is the basin of attraction of a stable gene expression pattern associated with a floral or meristematic identity. We apply this framework to the wild-type network, three single homeotic mutants (*ap1*, *pi*, and *ag*), and three double mutants (*ap3–pi*, *ag–pi*, and *ap1–ag*). For each genotype, we quantify landscape organization using complementary descriptors of basin structure, convergence depth, fate diversity, dominance, inequality, and Jensen–Shannon divergence from wild type. The resulting landscapes reveal distinct modes of mutant-induced deformation. The *ap1* mutant restricts fate accessibility and concentrates trajectories into dominant basins, whereas *pi* eliminates B-function-dependent identities while largely preserving global basin organization. In contrast, *ag* increases effective fate diversity despite the loss of reproductive identity, reflecting defective meristem termination and convergence to *WUS*-associated states. Double mutants exhibit non-additive deformation: *ap3–pi* is indistinguishable from *pi* across all reported descriptors, consistent with logical saturation of the AND-like B-function module, whereas *ag–pi* and *ap1–ag* produce distinct redistributions of fate accessibility. The framework recovers canonical floral identities and experimentally observed mutant phenotypes while treating the epigenetic landscape as a finite, computable object determined by regulatory logic. The framework thus provides a topology-based description of developmental robustness, epistasis, and mutant-induced landscape deformation.

## Introduction

Understanding the process of flower development in angiosperms remains a central problem in developmental biology. The emergence of distinct floral organs from initially undifferentiated tissues requires the coordinated action of gene regulatory networks (GRNs), whose collective dynamics give rise to robust and reproducible morphological patterns while allowing alternative developmental outcomes under genetic perturbations (Davidson and Erwin 2006). This apparent coexistence of robustness and flexibility has long been conceptualized through Waddington’s epigenetic landscape (Waddington 1942), and more recently formalized in terms of multistability, attractors, and state-space structure of regulatory networks (Waddington 2014; Huang 2009, 2012; Ferrell 2012).

In previous work (Pérez-Buendía et al. 2022), we introduced a discrete dynamical systems framework to model the GRN controlling floral morphogenesis in *Arabidopsis thaliana*. By representing the global dynamics of the network as an Epigenetic Forest (a collection of rooted in-arborescences induced by the state transition graph), we proposed a combinatorial analogue of Waddington’s epigenetic landscape. In this representation, each tree corresponds to the basin of attraction of a stable gene expression pattern, providing a structured and exhaustive view of differentiation trajectories toward specific floral or meristematic fates. In contrast to potential-based or quasi-energy formulations of the epigenetic landscape, our approach represents the landscape explicitly as a discrete, finite state-space object determined by the regulatory logic of the GRN. The Boolean update rules used here are those of Espinosa-Soto et al. (2004); one of the authors (Y.C.-P.) participated in that line of modeling.

This approach belongs to a long-standing line of work that uses basins of attraction of Boolean networks to interpret cell states and gene-regulatory dynamics, from Kauffman’s seminal random Boolean network ensembles (Kauffman 1969) and Albert and Othmer’s analysis of the *Drosophila* segment-polarity network (Albert and Othmer 2003) to systematic studies of how rule perturbations reshape state-transition graphs (Xiao and Dougherty 2007), ensemble-level entropy of basins (Krawitz and Shmulevich 2007), Hamming-neighbor measures of dynamic stability for cell states (Joo et al. 2018), and computational tools for basins and commitment sets in asynchronous Boolean networks (Klarner et al. 2020). In parallel, recent work has revisited Waddington’s landscape from a dynamical-systems perspective, including data-driven landscape reconstructions from single-cell measurements (Cislo et al. 2025). Our framework differs from these in that we organize the comparison along an explicit, multidimensional descriptor space (introduced in Section 3.4) and apply it to biologically grounded loss-of-function clamps in a well-characterized GRN.

The present work extends that framework in three ways. First, we apply the Epigenetic Forest to single and double homeotic mutants (*ap1*, *pi*, *ag* and *ap3–pi*, *ag–pi*, *ap1–ag*), enabling a systematic comparison of landscape deformation across genotypes. Second, we introduce a set of global quantitative descriptors–basin entropy, effective fate number, dominance measures, convergence depth, and Jensen–Shannon divergence from wild type–that were not used in the original formulation (Pérez-Buendía et al. 2022); these allow deformation modes to be compared on a common scale. Third, we formalize a notion of $\varepsilon$-indistinguishability in descriptor space and use it to quantify epistasis at the level of the full landscape, including logical saturation of the B-function module in *ap3–pi* relative to *pi*. Together, these extensions support a quantitative and topology-based interpretation of robustness and epistasis in the floral GRN.

We use these extensions to investigate how homeotic mutations reshape the structure of the Epigenetic Forest. These mutations are well-characterized perturbations of key transcription factors and provide a biologically grounded setting for examining how mutational robustness and access to alternative developmental outcomes are reflected in the global topology of the landscape. We focus on three classical single mutants of *Arabidopsis thaliana*, *APETALA1* (*ap1*), *PISTILLATA* (*pi*), and *AGAMOUS* (*ag*), and on three representative double mutants, *ap3–pi*, *ag–pi*, and *ap1–ag*. These perturbations probe distinct modes of deformation: loss of floral commitment (A), loss of organ identity specification (B), loss of meristem termination (C), and their combinations.

This work provides an exhaustive quantitative comparison of wild-type and mutant epigenetic landscapes. The descriptors introduced above characterize how each perturbation deforms fate accessibility, canalization, and fate diversity. The analysis shows that distinct mutations induce different deformation regimes, including restricted accessibility, identity collapse, and increased indeterminacy, while double mutants reveal strongly non-additive epistatic effects at the level of landscape structure. Although illustrated here for the floral GRN, the framework applies to any Boolean network with finite deterministic dynamics.

The paper is organized as follows. Section 2 summarizes the biological background: floral organogenesis, the ABC model, and the role of homeotic mutations. Section 3 presents the discrete dynamical system, the construction of the Epigenetic Forest, and the incorporation of mutants. Section 4 analyzes single and double mutants qualitatively (attractor structure, accessibility) and quantitatively (basin entropy, dominance, convergence, Jensen–Shannon divergence). Section 5 discusses biological and mathematical implications, modeling assumptions, and relation to other landscape-based approaches. Section 6 summarizes the main findings and the interpretation of the epigenetic landscape as a computable, topology-driven object.

## Biological Background

This section summarizes the biological elements needed for the model: floral organ organization, the ABC specification logic, the Arabidopsis floral GRN, and the homeotic mutants analyzed below.

### Flower Development and the ABC Model

Across angiosperms, flower architecture is commonly organized into four concentric whorls that give rise, from outside to inside, to sepals, petals, stamens, and carpels, each with distinct organ identity and function. In *Arabidopsis thaliana*, this broad architectural pattern is realized in a highly stereotyped form: a typical flower consists of four sepals, four petals, six stamens, and two fused carpels. Its genetic tractability and fully sequenced genome have made *Arabidopsis* a central system for studying flower development, and its regular floral architecture provides a useful reference for connecting ABC organ-identity logic with attractor structure in the floral GRN (Coen and Meyerowitz 1991; Alvarez-Buylla et al. 2010; Initiative 2000).

At the core of this process lies the floral meristem, a population of undifferentiated cells capable of sustained proliferation and differentiation. Within this tissue, the GRN integrates spatial and temporal cues to activate specific genetic programs, giving rise to organ primordia at well-defined positions. The reproducibility of floral architecture across individuals reflects the robustness of this regulatory logic, whereas its response to genetic perturbations reveals the extent to which the network buffers mutations or permits alternative developmental outcomes (Wagner 2005; Arjan et al. 2003; Phillips 2008).

The ABC model provides a parsimonious conceptual framework to describe how combinations of homeotic gene activities specify floral organ identity. In this model, class A genes act in the outer whorls to specify sepals; combined A and B activity specifies petals; B and C activity together specifies stamens; and class C activity alone specifies carpels in the innermost whorl. This combinatorial logic is summarized in Table 1.

**Table 1:** Organ identity determined by combinations of ABC gene expression (1 for active, 0 for inactive)

A	B	C	Organ
1	0	0	Sepal
1	1	0	Petal
0	1	1	Stamen
0	0	1	Carpel

Subsequent formulations have extended the ABC model by incorporating the SEPALLATA E-class genes (Pelaz et al. 2000), the floral quartet model of MADS-box transcription factor complexes (Theißen and Saedler 2001), comprehensive interaction maps of MADS-box partners (Stefan De Folter et al. 2005), and the broader ABCDE synthesis (Causier et al. 2010). Nevertheless, the ABC model remains a foundational paradigm in floral developmental biology and serves here as a conceptual baseline for interpreting how GRN dynamics give rise to stable organ identities and how these identities are altered under homeotic perturbations.

### The Gene Regulatory Network of *Arabidopsis thaliana*

While the ABC model provides a phenomenological framework for understanding floral organ identity, the underlying molecular mechanisms are governed by a complex gene regulatory network (GRN) composed of interacting transcription factors. This network integrates spatial and temporal signals within the floral meristem to regulate the activation and repression of key developmental genes, thereby constraining cell fate decisions during flower development.

In *Arabidopsis thaliana*, the core floral GRN involves homeotic genes such as *APETALA1 (AP1)*, *APETALA2 (AP2)*, *APETALA3 (AP3)*, *PISTILLATA (PI)*, *AGAMOUS (AG)*, and members of the *SEPALLATA (SEP)* family, together with regulatory factors including *LEAFY (LFY)*, *TERMINAL FLOWER1 (TFL1)*, *EMBRYONIC FLOWER1 (EMF1)*, *FRUITFULL (FUL)*, and *WUSCHEL (WUS)*. These genes interact through activating and repressing regulatory relationships that form feedback and feedforward motifs essential for maintaining meristem identity and orchestrating organ differentiation.

**Fig. 1:**
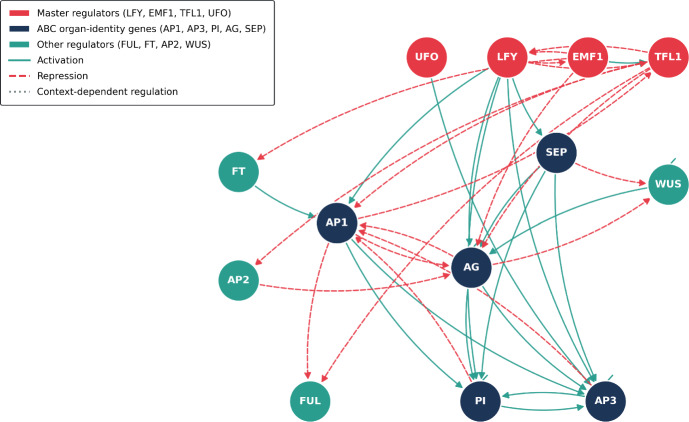
Gene regulatory network underlying floral development in *Arabidopsis thaliana*. Nodes are colored by functional class (master regulators, organ identity genes, other regulators). The network is directed: every edge has an explicit head (target gene) and tail (regulator), and represents a regulatory interaction derived from the Boolean rules. Edge styles encode the sign of the interaction: solid arrows indicate activation, dashed arrows indicate repression, and gray edges indicate context-dependent regulation (interactions whose sign or target depend on the state of other nodes; see Appendix 1 for rule definitions). Self-loops represent autoregulation. This network defines the deterministic dynamics used to construct the Epigenetic Forests analyzed in this work (Color figure online)

The gene regulatory network considered in this work is shown in Figure 1. Each node represents a gene, and directed edges correspond to experimentally established regulatory interactions, either activation or repression. The system is encoded as a discrete dynamical model by assigning Boolean variables to each gene (active or inactive) and defining logical update rules that determine the future state of the system based on its current configuration.

The Boolean update functions are those of the previously validated floral GRN model of *Arabidopsis thaliana* (Alvarez-Buylla et al. 2010). The complete set of 13 update functions is provided in Appendix 1. In the present work, this regulatory logic is used as a fixed dynamical substrate: our analysis focuses on the global state transition graph induced by the Boolean dynamics and on how its attractor basins are reorganized under homeotic perturbations.

This Boolean representation enables an exhaustive exploration of the full state space of the GRN. Each configuration of gene activity corresponds to a possible cellular state, and the induced state transition graph captures all admissible developmental transitions. Stable attractors of this graph can be interpreted as differentiated floral or meristematic identities, whereas transient states correspond to intermediate or undecided configurations.

This formalism allows genetic mutations to be incorporated as structural perturbations of the GRN. For example, a loss-of-function mutation in *AG* is modeled by fixing the corresponding node to an inactive state, effectively removing class C activity from the network. The resulting changes in the global structure of the state transition graph, including the number and nature of attractors, can then be analyzed using graph-theoretic methods.

In the following section, we introduce the formal construction of the Epigenetic Forest, which organizes the state transition graph into a collection of rooted trees and provides a discrete analogue of Waddington’s epigenetic landscape.

### Homeotic mutants and their phenotypic effects

Homeotic mutations are genetic perturbations that transform one organ type into another by disrupting the spatial and combinatorial expression of floral identity genes. These mutants played a foundational role in the formulation of the ABC model of flower development (Coen and Meyerowitz 1991; Bowman et al. 1991), and remain a central experimental system for dissecting the functional architecture of the gene regulatory network (GRN) controlling floral organ specification in *Arabidopsis thaliana*. By analyzing the phenotypic consequences of targeted gene knockouts, causal relationships between regulatory gene activity, spatial patterning, and organ identity have been firmly established.

From a modeling perspective, homeotic mutants constitute well-defined and biologically interpretable perturbations of the GRN. They correspond to discrete, localized disruptions of regulatory logic and therefore provide a natural testbed for assessing how structural changes in the network reorganize global developmental dynamics and differentiation trajectories.

**Fig. 2:**
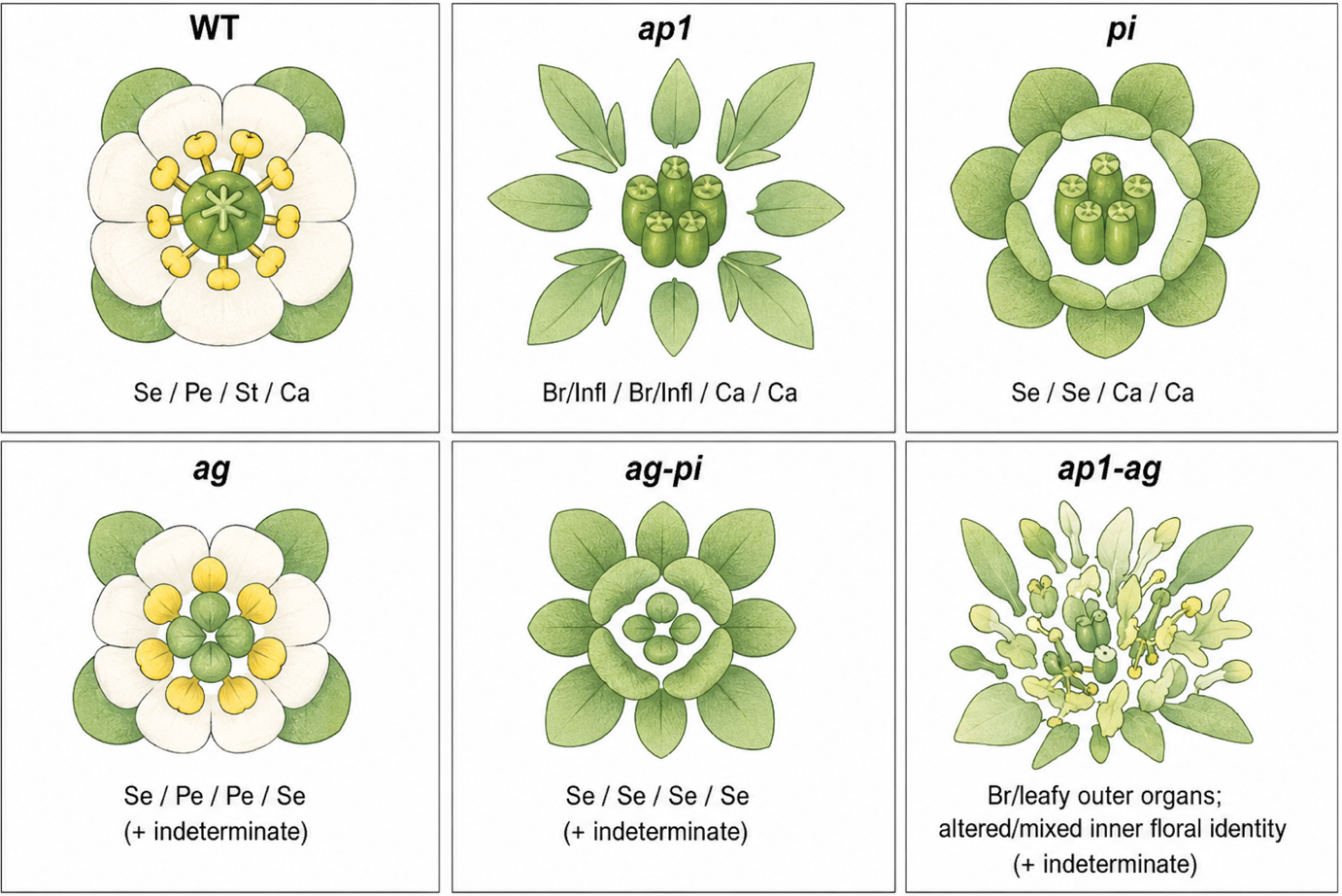
Schematic summary of canonical wild-type and mutant floral phenotypes considered in this work. Wild type (WT) displays the canonical sequence of sepals, petals, stamens, and carpels. The *ap1* mutant shows defective floral commitment, with bract- or inflorescence-associated outer structures and altered inner organ specification. The *pi* mutant lacks B-function, resulting in sepals in the second whorl and carpels in the third whorl. The *ag* mutant lacks C-function, producing repeated perianth organs and indeterminate floral growth. The *ag–pi* double mutant shows sepal-like organs across whorls together with indeterminacy. The *ap1–ag* double mutant exhibits bract- or leafy outer organs and altered or mixed inner floral identity, also with indeterminate growth. The *ap3–pi* double mutant is not shown separately because loss of *PI* already abolishes B-function in this Boolean model, making its canonical organ-identity outcome equivalent to that of *pi*. Schematic based on canonical phenotypes described in Bowman et al. (1991); Coen and Meyerowitz (1991); Alvarez-Buylla et al. (2010) (Colour figure online)

The three classical single mutants relevant to the present analysis are *ap1*, *pi*, and *ag*, corresponding to loss of A-, B-, and C-function in *Arabidopsis thaliana*, respectively. The canonical organ-identity outcomes of these mutants are summarized in Table 2 and visualized schematically, together with the double mutants analyzed below, in Figure 2.

**Table 2:** Canonical organ identity outcomes in wild-type and selected homeotic mutants based on ABC gene expression. Mutants *ap1*, *pi*, and *ag* correspond to loss of A-, B-, and C-function, respectively. “Infl.” = Inflorescence-associated

Whorl	Wild Type	*ap1*	*pi*	*ag*
1	Sepal (A)	Bract-like / Infl.	Sepal (A)	Sepal (A)
2	Petal (A+B)	Bract-like / Infl.	Sepal (A)	Petal (A+B)
3	Stamen (B+C)	Carpel (C)	Carpel (C)	Petal (A+B)
4	Carpel (C)	Carpel (C)	Carpel (C)	Sepal (A)

Within our framework, these homeotic mutants are incorporated by fixing the corresponding gene nodes to an inactive state in the Boolean GRN. This intervention alters the global structure of the state transition graph, leading to changes in the number, identity, and organization of attractors. The resulting reconfiguration of differentiation pathways is captured and analyzed through the corresponding Epigenetic Forests, allowing a direct connection between classical mutant phenotypes and global landscape topology.

The next section introduces the discrete dynamical system underlying this construction and formalizes the Epigenetic Forest representation used to compare wild-type and mutant developmental landscapes.

## The Discrete Dynamical System and the Epigenetic Forest

To investigate the developmental dynamics underlying floral organ formation and their reorganization under genetic perturbations, we model the gene regulatory network (GRN) of *Arabidopsis thaliana* as a discrete dynamical system. This formulation enables an exhaustive exploration of the space of gene expression configurations, the identification of differentiation trajectories, and the reconstruction of a discrete analogue of Waddington’s epigenetic landscape within a mathematically rigorous and computationally tractable framework.

In this section, we first describe how the GRN is encoded as a Boolean dynamical system and how its associated state transition graph is constructed. We then introduce the notion of an Epigenetic Forest, obtained by decomposing the transition graph into basins of attraction rooted at stable fixed points. This representation provides a global and structured view of the differentiation landscape induced by the network dynamics. Finally, we describe how homeotic mutations are incorporated into the model and compare their developmental behaviors with the wild-type flower.

### Modeling the GRN as a Discrete Dynamical System

We represent the gene regulatory network (GRN) as a Boolean network with $n = 13$ nodes, each corresponding to a gene $g_i$ that can be either active (ON, 1) or inactive (OFF, 0). The state of the system at time *t* is given by a binary vector

$$ x(t) = (x_1(t), \ldots , x_{13}(t)) \in \{0,1\}^{13}, $$

where $x_i(t)$ denotes the expression state of gene $g_i$.

The temporal evolution of the network is governed by a collection of Boolean update functions $f_i: \{0,1\}^{13} \rightarrow \{0,1\}$, each encoding the regulatory inputs acting on gene $g_i$. The update rules used here are those of Espinosa-Soto et al. (2004) (The Plant Cell, 2004). Assuming synchronous updates, the global dynamics are defined by the map

$$ F : \{0,1\}^{13} \rightarrow \{0,1\}^{13}, \qquad F(x(t)) = \bigl ( f_1(x(t)), f_2(x(t)), \ldots , f_{13}(x(t)) \bigr ). $$

This formulation induces a deterministic discrete-time dynamical system evolving on the finite state space

$$ \mathcal {S} = \{0,1\}^{13}, $$

which contains $2^{13} = 8192$ possible gene expression configurations. The associated state transition graph $\mathcal {G}$ is defined by taking each state $x \in \mathcal {S}$ as a vertex and introducing a directed edge from *x* to *F*(*x*).

States satisfying $F(x) = x$ are fixed points of the dynamics and correspond to stable gene expression patterns. In the context of floral development, these fixed points are interpreted as differentiated cellular fates.

Under wild-type conditions, the GRN admits exactly ten fixed points. Each of these fixed points represents a terminal developmental state, and together they partition the state space into disjoint basins of attraction. As a result, every initial condition in $\mathcal {S}$ converges in finite time to one of these ten attractors.

In the next subsection, we show how the structure of the transition graph $\mathcal {G}$ naturally defines a discrete differentiation landscape: the Epigenetic Forest, where each basin of attraction forms a rooted tree associated with a specific floral identity.

### Epigenetic Forests and Transition Graph Construction

The Epigenetic Forest provides a structured representation of the differentiation landscape induced by the dynamics of the GRN. It is derived from the state transition graph $\mathcal {G}$ introduced in the previous subsection, where each vertex corresponds to a gene expression state $x \in \{0,1\}^{13}$ and each directed edge represents a transition $x \rightarrow F(x)$ under synchronous updates.

Due to the deterministic nature of the dynamics, the state transition graph is a functional digraph: each state has out-degree one. In such graphs, every connected component contains exactly one directed cycle, with in-arborescences feeding into it. Exhaustive enumeration of the $2^{13}$ states shows that, under the synchronous update scheme used here, all periodic orbits are fixed points. Thus, the state transition graph decomposes into basins of attraction rooted at fixed points.

Consequently, every state in $\mathcal {S}$ converges in finite time to a fixed-point attractor. Each basin forms an in-arborescence, a directed tree whose edges follow the dynamics toward the attractor, with out-degree one at every non-attractor state and the fixed point as its root. The collection of these basin trees constitutes the Epigenetic Forest. In what follows, we use the term *rooted tree* in this functional-digraph sense.

The forest is constructed by exhaustively evaluating the global update function *F* on all $2^{13}$ states in $\mathcal {S}$. For each initial condition, the corresponding trajectory is iterated until convergence to a fixed point, thereby assigning the state to a unique basin of attraction. This procedure yields a complete decomposition of the transition graph into rooted trees encoding all possible differentiation trajectories supported by the GRN dynamics.

**Fig. 3:**
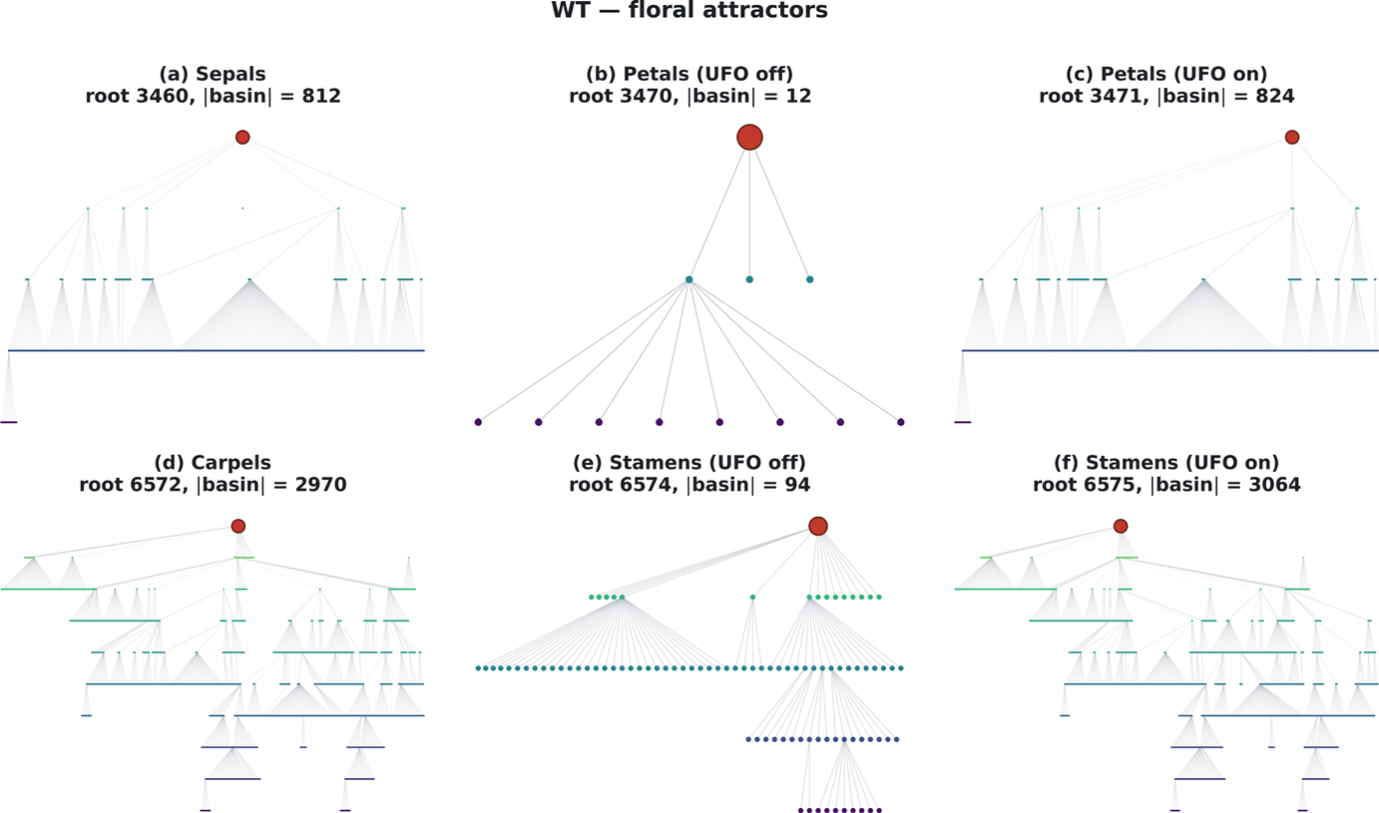
Epigenetic Forest trees corresponding to floral organ identities in the wild-type GRN. **(a)** Sepals, **(b)** Petals with *UFO* ON, **(c)** Petals with *UFO* OFF, **(d)** Stamens with *UFO* ON, **(e)** Stamens with *UFO* OFF, **(f)** Carpels. Each tree represents the basin of attraction of a stable gene expression pattern associated with a specific floral organ

Each fixed point is annotated with a biological identity based on the expression pattern of key marker genes, namely *AP1*, *PI*, *AG*, *SEP*, and *UFO*. This classification results in ten terminal identity classes: four inflorescence-related attractors and six floral organ attractors. Petal- and stamen-specifying ABC configurations each occur in two stable variants that differ in the state of *UFO*. These variants are distinct fixed points of the Boolean dynamics, although they map to the same organ identity at the phenotypic level. Accordingly, basin-level summaries retain the two variants, whereas organ-level summaries aggregate them when comparing basin distributions across organ-identity classes. Biologically, these two fixed-point variants are not interpreted as distinct mature organ identities. Rather, they represent stable configurations of the Boolean dynamics that are compatible with the same morphological organ class, because *UFO* expression is spatially and temporally restricted during second- and third-whorl patterning (Espinosa-Soto et al. 2004; Alvarez-Buylla et al. 2010). Thus, *UFO*-on and *UFO*-off fixed points are retained as distinct dynamical attractors in basin-level summaries, but are aggregated as the same organ identity in organ-level summaries Fig. 3.

**Fig. 4:**
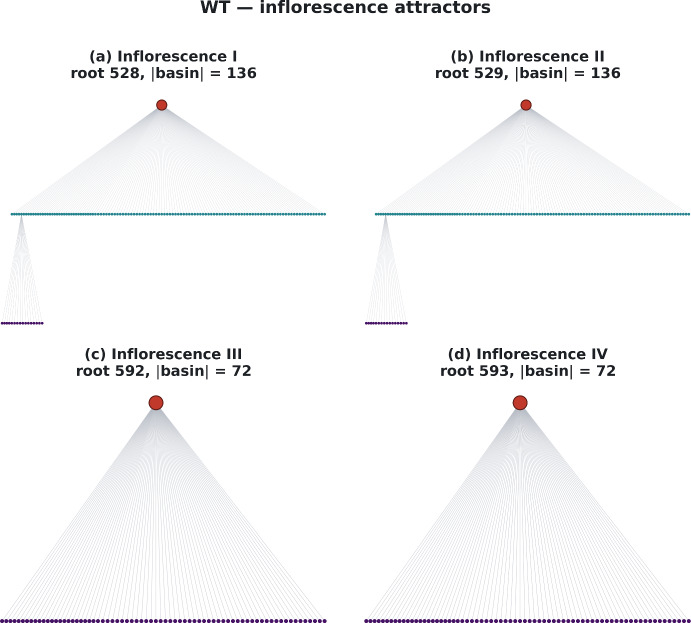
Epigenetic Forest trees corresponding to the four inflorescence attractors. **(a)** Inflorescence type I, **(b)** Type II, **(c)** Type III, **(d)** Type IV. These attractors correspond to meristematic or undifferentiated cellular states, typically characterized by sustained *TFL1* expression and repression of floral identity genes

Beyond cataloguing terminal cell fates, the Epigenetic Forest captures the combinatorial logic of differentiation imposed by the GRN dynamics. By explicitly encoding all possible gene expression trajectories and their convergence to stable floral or meristematic states, this structure reveals both the diversity of attainable cell identities and the topological organization of the underlying regulatory landscape. As such, it provides a natural reference framework for systematic comparisons between wild-type and genetically perturbed networks.

#### Relation to Basin Entropy and Operational Notions of Canalization

In the present framework, canalization is quantified through properties of the state-transition graph. Two aspects are relevant: the concentration of trajectories into a small number of dominant attractor basins, and the speed with which trajectories reach terminal fates. These graph-theoretic descriptors are related to the classical biological notion of canalization as developmental buffering (Arjan et al. 2003), but they are computed here directly from basin sizes and convergence depths.

Because the dynamics are deterministic and synchronous, each state has a unique successor. The resulting forest encodes trajectory convergence and basin organization, but not the Hamming-neighborhood proximity between different basins. Measures of basin adjacency or perturbational robustness on the Boolean hypercube therefore require a separate neighbor-aware analysis (Joo et al. 2018).

The associated descriptors summarize the geometry of the state-transition graph induced by the synchronous map *F*. We organize this geometry along three operational axes:

- **Fate-level structural canalization** (*Is fate accessibility concentrated?*): quantified by basin-size descriptors, including $p_{\max }$, the fraction of states belonging to the largest basin of attraction; $p_{\textrm{top2}}$, the cumulative fraction belonging to the two largest basins; the Gini coefficient *G*, and the basin entropy *H*. Low *H* and high $p_{\max }$ indicate concentration of accessibility into a small number of dominant fates.
- **Temporal canalization** (*How quickly is fate committed?*): captured by mean and maximum convergence times $\langle L\rangle$ and $\max _x L(x)$ over the full state space.
- **Fate diversity** (*How many comparable fates are present?*): captured by the effective number of fates $N_{\textrm{eff}}=2^{H}$.

These axes are independent: a landscape can have short convergence times (strong temporal canalization) and at the same time many comparably sized basins (low structural canalization, high fate diversity). The *ap1–ag* mutant illustrates this combination (Section 4.2).

The Shannon entropy of basin sizes,

$$ H=-\sum _i p_i\log _2 p_i, $$

is therefore used as a fate-distribution descriptor, in the spirit of basin entropy in random Boolean network ensembles (Krawitz and Shmulevich 2007). Together, the descriptors introduced above provide complementary measures of fate concentration, fate diversity, and dynamical depth across genotypes.

### Incorporating Homeotic Mutants into the GRN

Homeotic mutants perturb floral development by disrupting the identity and spatial arrangement of organs through the loss of key regulatory functions. Within our Boolean framework, such mutations are implemented as loss-of-function conditions by fixing the state of specific genes to 0 (clamping). Formally, let $F:\{0,1\}^n \rightarrow \{0,1\}^n$ denote the wild-type update map, and let $\textrm{clamp}_{j,0}$ denote the operator that sets the *j*-th coordinate to 0. For a loss-of-function mutation in gene *j*, we define the mutant dynamics by the output-clamped map

$$ \widetilde{F}^{(j,0)}(x) := \textrm{clamp}_{j,0}\big (F(x)\big ). $$

Iterating $\widetilde{F}^{(j,0)}$ on the full state space yields a modified state transition graph $\mathcal {G}^{(\textrm{mut})}$ and Epigenetic Forest $\mathcal {F}^{(\textrm{mut})}$ with altered attractor set and basin structure relative to wild type. All reported landscape metrics for mutants are computed for this dynamics on the full state space $\{0,1\}^n$.

In this study, we focus on the three classical single homeotic mutants *ap1*, *pi*, and *ag* of *Arabidopsis thaliana* (loss of A-, B-, and C-function, respectively), whose canonical phenotypes were summarized in Section 2.3 (Table 2). For each mutant condition, we reconstruct the corresponding Epigenetic Forest and identify the modified set of attractors. Exhaustive enumeration shows that, under the synchronous update scheme, all mutant trajectories converge to fixed points, with no periodic orbits of length greater than one. Comparing these forest topologies with the wild-type landscape allows us to quantify how homeotic perturbations redirect developmental trajectories and reorganize fate accessibility.

Beyond single-gene perturbations, the combinatorial structure of the floral GRN naturally raises the question of how simultaneous disruptions of multiple regulatory functions reshape the epigenetic landscape. Single mutants probe the contribution of individual nodes, whereas double mutants reveal whether landscape deformations combine additively or produce non-linear interactions encoded in the network architecture. Within the Epigenetic Forest framework, these combined perturbations expose epistatic effects as changes in global landscape structure, including descriptor-level equivalence, basin redistribution, and altered convergence dynamics.

In the following sections, we analyze each mutant configuration, including both single and double perturbations, by characterizing changes in attractor composition, forest topology, and differentiation trajectories, and by relating these structural deformations to the corresponding developmental phenotypes.

### Quantitative descriptors of epigenetic landscapes

Beyond the identification of attractors and their associated basins, a central objective of the Epigenetic Forest framework is to provide quantitative descriptors that summarize the global organization of the landscape induced by a discrete gene regulatory network. These descriptors are defined directly on the full state transition graph of the synchronous Boolean dynamics and are independent of any particular biological condition or perturbation.

Let $\mathcal {A}$ denote the set of fixed-point attractors of the system, and let $\mathcal {B}_i \subset \{0,1\}^n$ be the basin of attraction of attractor $i \in \mathcal {A}$. We define the basin fraction

$$ p_i := \frac{|\mathcal {B}_i|}{2^n}, $$

which represents the probability that a uniformly sampled initial condition converges to attractor *i*.

*Basin entropy and effective number of fates.* The Shannon entropy of the basin distribution,

$$ H = -\sum _{i \in \mathcal {A}} p_i \log _2 p_i, $$

quantifies how evenly the state space is partitioned among terminal fates. Low entropy indicates a landscape dominated by a small number of basins, whereas higher entropy corresponds to a more homogeneous accessibility of attractors. Entropy-based measures have been previously proposed to quantify uncertainty and basin structure in dynamical systems (Daza et al. 2016).

For interpretability, we define the effective number of fates

$$ N_{\textrm{eff}} = 2^{H}, $$

which corresponds to the number of equally sized basins that would produce the same entropy. This quantity provides a scale-independent measure of fate diversity.

*Dominance and inequality of basin occupancy.* Entropy alone does not fully capture the presence of dominant fates. We therefore characterize basin dominance using the largest basin fraction

$$ p_{\max } = \max _i p_i, $$

and the cumulative weight of the two largest basins,

$$ p_{\textrm{top2}} = p_{(1)} + p_{(2)}, $$

where $p_{(1)} \ge p_{(2)} \ge \dots$ denotes the ordered basin fractions.

To quantify inequality across all basins, we compute the Gini coefficient, a standard measure of inequality in probability distributions (Frank 2011).

Let $\mathcal {A}=\{1,\dots ,m\}$ denote the set of attractors and $p_i = |\mathcal {B}_i|/2^{n}$ the normalized basin fraction of attractor *i*, so that $\sum _{i=1}^{m} p_i = 1$. The Gini coefficient is then defined as

$$ G = \frac{1}{2m} \sum _{i=1}^{m} \sum _{j=1}^{m} |p_i - p_j|. $$

The Gini coefficient ranges from $G=0$ for perfectly equal basin sizes to $G \rightarrow 1$ for extreme dominance by a single attractor.

*Convergence depth.* In addition to basin occupancy, the Epigenetic Forest encodes dynamical information about the trajectories leading to terminal states. For each initial condition $x \in \{0,1\}^n$, let *L*(*x*) denote the number of synchronous updates required to reach its terminal attractor. We summarize convergence depth by the mean convergence time

$$ \langle L \rangle = \frac{1}{2^n} \sum _x L(x), $$

and the maximum convergence time $\max _x L(x)$. These quantities characterize how rapidly the system commits to a fate and whether differentiation trajectories are shallow or deep in the state transition graph. Mean and maximum convergence times are reported for all genotypes in Section 4 (Tables 3 and 6) and quantify the speed of fate commitment under synchronous dynamics.

*Landscape-level divergence.* To compare epigenetic landscapes associated with different network parameterizations or perturbations, we represent each landscape as a probability distribution over coarse-grained fate classes. Each attractor is assigned to one of five classes–sepal, petal, stamen, carpel, or inflorescence–based on the expression pattern of organ-identity genes (*AP1*, *PI*, *AG*, *SEP*) consistent with the ABC model (Table 1); the probability mass of each class is the sum of basin fractions of attractors in that class. Given two such distributions *P* and *Q*, we define their Jensen–Shannon divergence (Endres and Schindelin 2003)

$$ \textrm{JS}(P,Q) = \tfrac{1}{2} D_{\textrm{KL}}(P \Vert M) + \tfrac{1}{2} D_{\textrm{KL}}(Q \Vert M), \quad M = \tfrac{1}{2}(P+Q), $$

where $D_{\textrm{KL}}$ denotes the Kullback–Leibler divergence. The Jensen–Shannon divergence is symmetric, bounded, and finite, making it well suited for quantifying global differences in fate accessibility between landscapes.

Together, these descriptors provide a quantitative characterization of epigenetic landscapes in terms of fate diversity, dominance, inequality, dynamical depth, and global divergence, enabling direct comparison between wild-type and perturbed networks.

## Results

We first examine how classical homeotic mutations alter attractor accessibility and biological identity. We then quantify basin organization, convergence, epistasis, and landscape divergence across single and double mutants. Throughout, results are reported from the exhaustive analysis of the full state transition graph; broader interpretation is deferred to the Discussion.

### Homeotic Mutants

#### The *ap1* Mutant: Disruption of Floral Initiation

The *APETALA1* (*AP1*) gene plays a central role in floral meristem identity and in the transition from inflorescence to floral development. In the wild-type network, *AP1* expression marks floral commitment and participates in regulatory interactions upstream of key floral identity genes such as *LFY*, *AG*, and *SEP*.

**Fig. 5:**
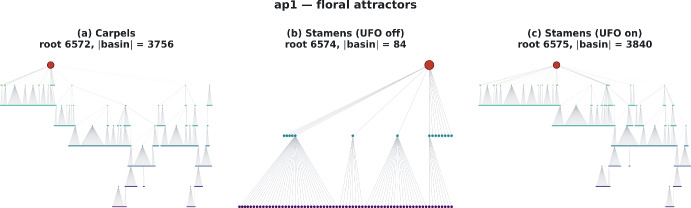
Transition trees rooted at wild-type floral attractors with active *AP1*. Panel (a): stamens with *UFO* ON; (b): stamens with *UFO* OFF; (c): carpels. All roots correspond to terminal floral attractors with stable *AP1* expression. Differences in tree width and depth reflect organ-specific basin structures in the epigenetic landscape

To assess the structural role of *AP1* within the Epigenetic Forest, we first examined the transition trees rooted at wild-type floral attractors with active *AP1* expression (Figure 5). These trees correspond to differentiated floral identities, including stamen variants distinguished by *UFO* activity and the carpel identity. These wild-type floral attractors are therefore consistent with successful floral specification following meristem commitment.

Beyond the presence of *AP1*, the topology of the corresponding trees reveals organ-specific structural differences. Trees associated with stamen identities (panels a and b) display variations in branching complexity and in-degree depending on *UFO* activity, whereas the carpel-associated tree (panel c) exhibits a broader, multi-tiered organization. This increased depth and branching heterogeneity suggests a more complex differentiation landscape for carpel fate. In all cases, inspection at the level of individual states confirms that *AP1* remains active at the roots, reinforcing its role as a stable component of floral attractor states.

**Fig. 6:**
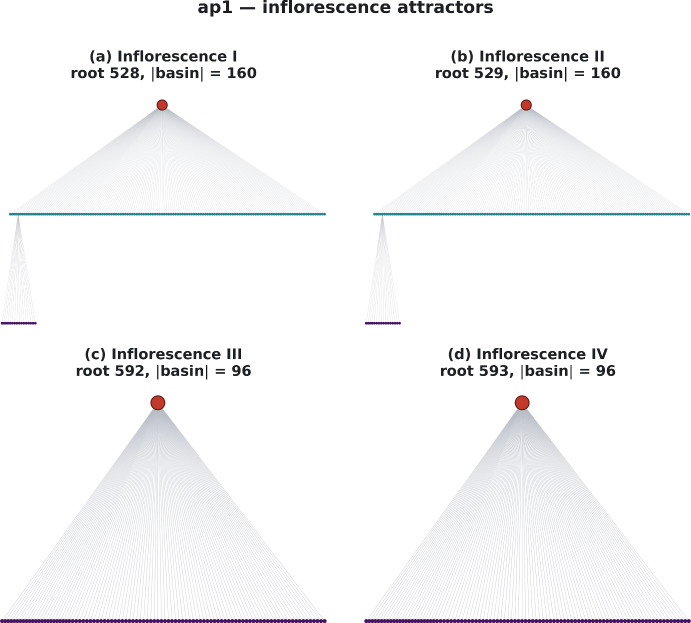
Inflorescence attractors under wild-type conditions. Panels (a)–(d) correspond to the four stable inflorescence states (pI1–pI4), all lacking *AP1* activity. The wide and shallow tree structures reflect a weakly committed meristematic landscape prior to floral commitment

In contrast, analysis of the inflorescence attractors under wild-type conditions (Figure 6) reveals a qualitatively different landscape. These trees, corresponding to states prior to floral commitment, exhibit complete absence of *AP1* activity. Structurally, they are characterized by wide, shallow geometries with limited hierarchical organization and relatively uniform branching patterns. This organization indicates rapid convergence toward meristematic fixed points, while the small basin masses of these attractors show that the meristematic class occupies only a limited portion of the wild-type state space.

Taken together, these results indicate that *AP1* affects both floral identity and landscape organization. Its activation is associated with the emergence of floral attractors and with a shift from a small-mass inflorescence domain toward floral basins with stronger fate-level structural canalization. In the Epigenetic Forest, *AP1* marks a transition between a meristematic regime, characterized by rapid convergence to sparsely occupied inflorescence attractors, and a more concentrated floral regime dominated by differentiated organ identities.

#### The *pi* Mutant: Collapse of Petal and Stamen Identity

**Fig. 7:**
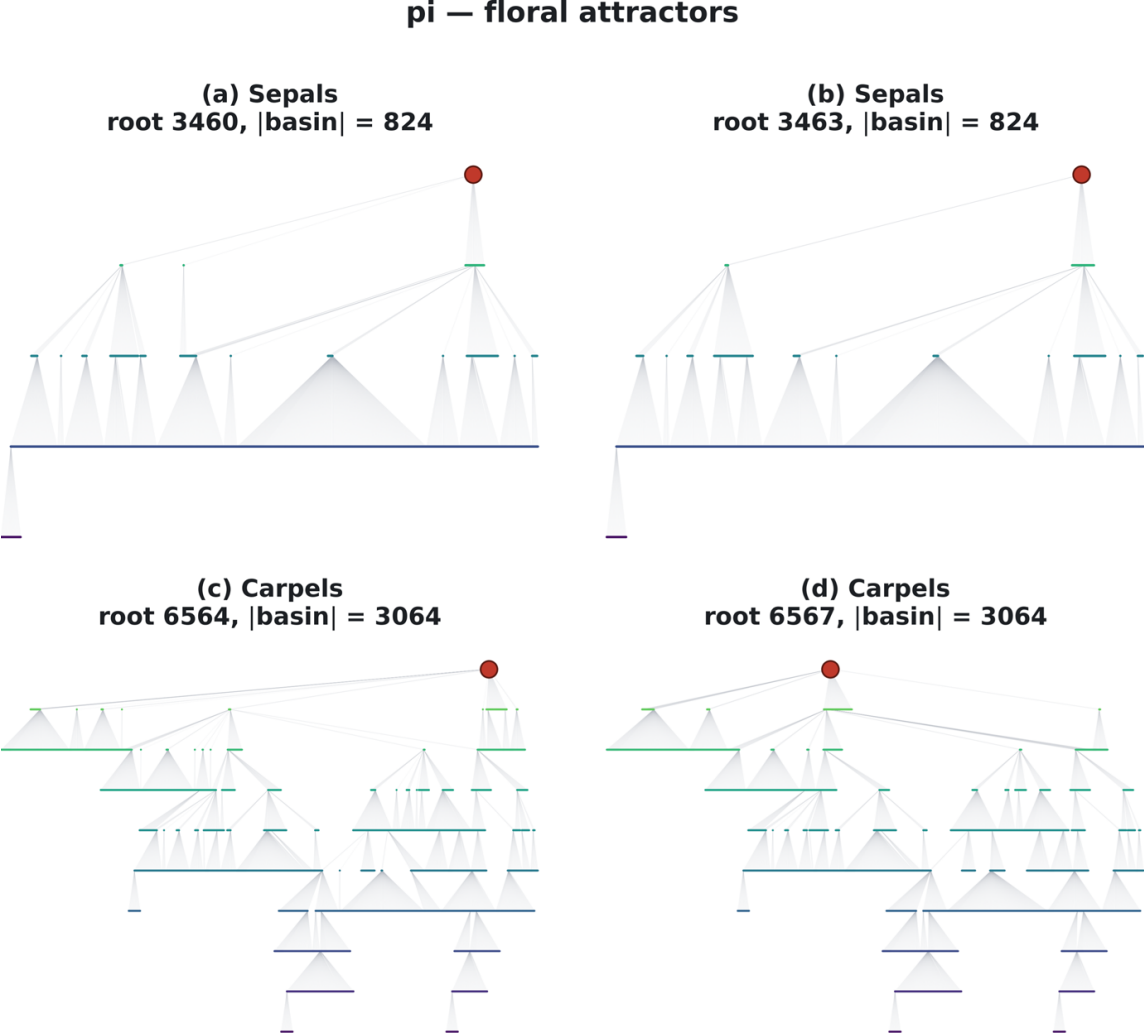
Epigenetic Forest trees of the *pi* mutant corresponding to floral attractors. Only sepal- and carpel-associated basins persist, reflecting the complete loss of B-function–dependent identities

The *PISTILLATA* (*PI*) gene encodes one of the two B-function partners, together with *AP3*. We simulated the *pi* loss-of-function condition by clamping the *PI* node to zero and analyzed the resulting attractors and their associated Epigenetic Forest. The mutant dynamics recover eight attractors (Figure 7): four corresponding to the wild-type inflorescence states ($\hbox {pI}_1$–$\hbox {pI}_4$), two corresponding to sepal identities, and two corresponding to carpel identities. No attractors corresponding to petals or stamens persist in the mutant landscape.

**Fig. 8:**
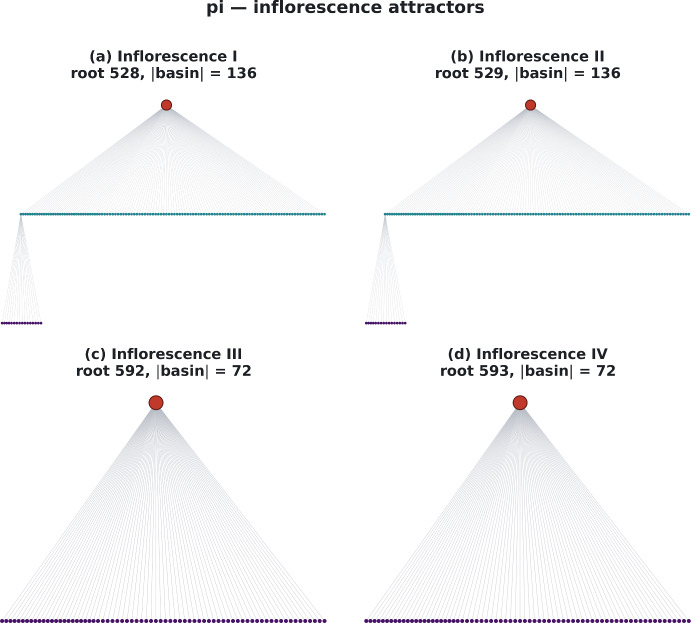
Epigenetic Forest trees corresponding to the four inflorescence attractors ($\hbox {pI}_1$–$\hbox {pI}_4$) in the *pi* mutant. Their persistence and wide, shallow topology indicate that loss of *PI* does not disrupt meristem maintenance or inflorescence identity

At the level of state representation, the disappearance of petal and stamen attractors can be traced directly to the loss of *PI* activity. In the wild-type network, petal and stamen attractors differ from sepal and carpel states by the activation of B-function genes. When *PI* is fixed to zero, these B-dependent configurations become dynamically inaccessible, and the corresponding regions of state space are redirected toward pre-existing A-only (sepal) or C-only (carpel) attractors Fig. 8.

Topologically, the *pi* mutant landscape contracts to a smaller set of terminal identities (eight attractors), with differentiation trajectories redirected toward outer (sepals) and inner (carpels) whorl fates and with B-function–dependent pathways eliminated. Basin mass is redistributed among the remaining sepal and carpel variants rather than collapsing onto a single dominant fate (see Section 4.2 for quantitative comparison). The *pi* mutant thus illustrates a distinct mode of deformation: loss of accessible identities without emergence of new ones, and redirection of state space toward canonical A-only and C-only fates. Within the Epigenetic Forest, this appears as the elimination of basins associated with petal and stamen identities, while the internal topology of the surviving sepal and carpel trees remains largely unchanged. This behavior is consistent with experimental observations that *pi* mutant organs adopt well-defined sepal or carpel expression programs, with no intermediate B-function attractors in the model.

#### The *ag* Mutant: Loss of Reproductive Identity

The *AGAMOUS* (*AG*) gene is the C-class regulator and also participates in floral meristem termination through repression of *WUSCHEL* (*WUS*), a key regulator of meristem maintenance. We simulated the *ag* loss-of-function condition by clamping the *AG* node to zero and analyzed how this perturbation affects attractor identity, *WUS* activity, and basin organization. Under wild-type conditions, *WUS* expression is downregulated following inner whorl specification through regulatory interactions involving the AG–SEP complex (Lenhard and Laux 2001). In contrast, experimental studies have shown that in *ag* mutants, *WUS* remains ectopically active in the floral center (Laux et al. 1996), preventing proper meristem termination.

**Fig. 9:**
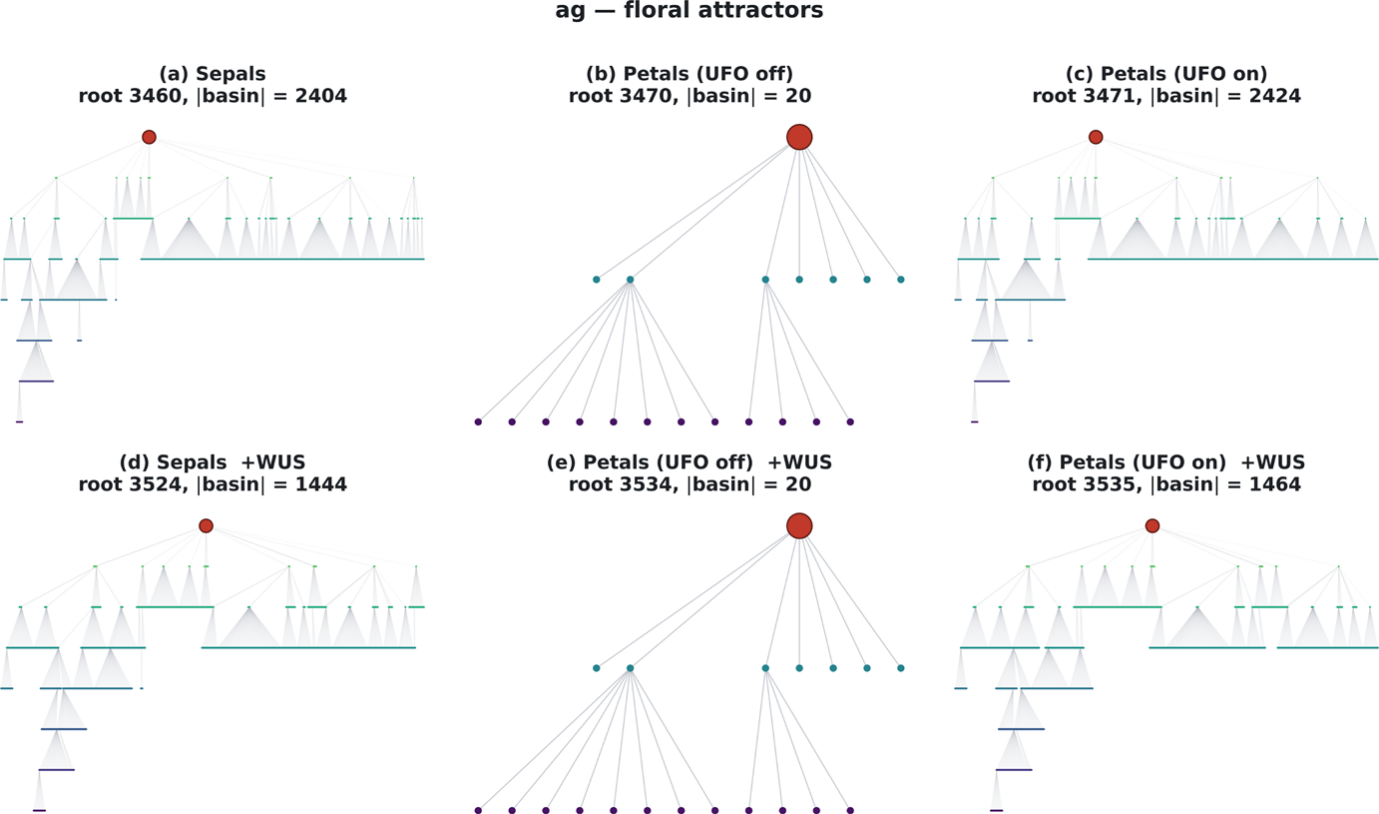
Epigenetic Forest of the *ag* mutant. Each tree represents the basin of attraction of a terminal state under the *AG*=0 condition. Panels (a)–(c) show wild-type floral identities with *WUS* repressed, whereas panels (d)–(f) display mutant attractors with persistent *WUS* activation. In reproductive-like identities (e, f) and in some perianth states, the *WUS* bit remains active, indicating failure of meristem termination

Figure 9 illustrates the Epigenetic Forests associated with floral organ identities under wild-type and *ag* mutant conditions, with particular emphasis on the expression state of *WUS* (bit 7). In the wild-type case (panels a–c), terminal attractors corresponding to sepals, petals, and stamens exhibit complete repression of *WUS*, consistent with floral meristem termination. In contrast, under *AG* loss-of-function (panels d–f), persistent or ectopic activation of *WUS* is observed in perianth- and reproductive-like attractors.

This regulatory alteration is reflected primarily in basin reassignment, with only limited changes in tree geometry. The wild-type and *ag*-mutant in-arborescences in Figure 9 have comparable overall depth and width, consistent with the convergence-time statistics in Table 3 ($\langle L \rangle = 3.16$ in *ag* versus 4.11 in wild type, $\max L = 7$ versus 9). Thus, the landscape signature of meristem-termination failure is the relocation of terminal states from *WUS*=0 attractors to nearby *WUS*=1 attractors, together with a redistribution of basin mass.

At the level of state representation, mutant attractors corresponding to inner whorl and perianth identities differ from their wild-type counterparts exclusively by the activation of *WUS*, while all organ-identity genes remain unchanged. Specifically, for attractors that in the wild-type network exhibit identical configurations of organ identity genes, loss of *AG* induces a consistent switch of the *WUS* bit from 0 to 1 .

This pattern is observed across multiple attractor classes, including petal-, stamen-, and carpel-associated states. The mutant terminal states therefore retain organ-identity configurations while preserving meristematic features through sustained *WUS* expression, consistent with the dual role of *AG* in organ specification and meristem termination.

To further characterize the effect of *AG* loss on meristematic states, we examined the transition trees rooted at the four inflorescence attractors ($\hbox {pI}_1$–$\hbox {pI}_4$). Compared with the wild type (Figure 4), these trees retain broadly similar depth and branching organization, indicating that *AG* loss has only limited effects on the internal topology of individual inflorescence basins. Its main effect at this level is a redistribution of basin mass: a larger fraction of the state space converges to inflorescence-like meristematic fixed points, consistent with persistent *WUS* activity and defective floral meristem termination.

**Fig. 10:**
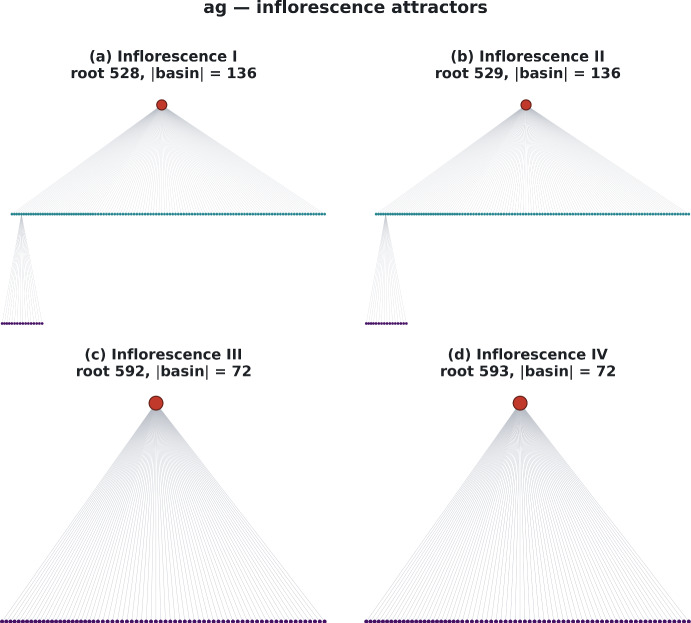
Transition trees rooted at the four inflorescence attractors under *AG* loss-of-function conditions. Each tree corresponds to a basin of attraction leading to a meristematic state with active *WUS*. The shallow depth and high symmetry reflect convergence to meristematic states with persistent *WUS* activity

In the Epigenetic Forest, this appears as increased global accessibility of meristematic attractors, together with the loss or redirection of reproductive attractors associated with *WUS*=0 toward nearby *WUS*=1 attractors. This redistribution provides the landscape-level signature of termination failure in the *ag* mutant Fig. 10.

Overall, the *ag* mutant induces a global reconfiguration of the epigenetic landscape, characterized by the loss of reproductive identity, persistent meristem activity, and a collapse of developmental termination. These results demonstrate how targeted perturbations of key regulatory nodes reshape both attractor identities and the global logic of differentiation encoded in the GRN dynamics.

### Quantitative landscape structure: basin size and convergence

We summarize global landscape structure using basin sizes and convergence-time statistics computed over the full $2^{13}$ state space, where basin sizes quantify fate accessibility and trajectory lengths capture the dynamical depth of differentiation.

Using the quantitative descriptors introduced in Section 3.4, we quantify how single-gene homeotic perturbations redistribute basin mass, dominance, and convergence depth across the full state space. This provides a global complement to the attractor-level analysis above.

*Wild-type reference landscape.* Under wild-type conditions, the epigenetic landscape exhibits a moderately heterogeneous basin structure, with basin entropy $H=2.129$ bits and an effective number of fates $N_{\textrm{eff}}=4.38$ (Table 3). Although ten attractors are present, basin occupancy is highly uneven: stamen and carpel identities dominate the landscape, together accounting for more than $70\%$ of the state space, as reflected in a large $p_{\max }=0.374$ and $p_{\textrm{top2}}=0.737$. The relatively high Gini coefficient ($G=0.655$) indicates strong fate-level structural canalization toward reproductive fates. The mean convergence time is $\langle L\rangle =4.11$ synchronous updates, with a maximum of $\max L=9$, providing the reference scale against which mutant landscapes are compared below.

This wild-type configuration provides a reference against which mutant-induced deformations can be interpreted.

*The ap1 mutant: increased dominance and reduced fate diversity.* Loss of *AP1* induces a pronounced collapse of basin diversity. Basin entropy drops sharply to $H=1.468$ bits, corresponding to an effective fate number of $N_{\textrm{eff}}=2.77$. This reduction reflects the elimination of all floral basins and the redirection of trajectories toward a small subset of remaining fates, primarily inflorescence-associated and inner-whorl identities.

Despite the reduction in fate repertoire, the landscape becomes more strongly canalized: the two largest basins together account for more than $92\%$ of the state space ($p_{\textrm{top2}}=0.927$), and the Gini coefficient remains high ($G=0.651$). Convergence times are shortened relative to wild type, indicating rapid commitment to non-floral states once floral initiation is blocked. Quantitatively, *ap1* therefore produces a landscape that is both structurally simplified and dynamically shallow.

*The pi mutant: redistribution without strong canalization.* In contrast, the *pi* mutant preserves a basin entropy comparable to wild type ($H=2.044$ bits, $N_{\textrm{eff}}=4.13$), despite the complete loss of petal and stamen identities. This reflects a redistribution of basin mass among the remaining sepal and carpel fates rather than a collapse onto a single dominant identity.

Although individual B-function–dependent basins disappear, their basin mass is redistributed across symmetry-related variants of A-only and C-only fates. As a result, $p_{\max }$ and $p_{\textrm{top2}}$ remain close to wild-type values, and inequality is moderately reduced ($G=0.590$). Mean and maximum convergence times remain similar to the wild-type landscape, indicating that loss of B-function constrains fate identity without substantially accelerating or deepening differentiation dynamics.

*The ag mutant: high diversity with persistent indeterminacy.* The *ag* mutant exhibits the highest basin entropy among single mutants ($H=2.283$ bits, $N_{\textrm{eff}}=4.87$), reflecting the emergence of additional attractors associated with perianth-like identities and persistent *WUS* activity. Unlike *ap1*, loss of *AG* does not reduce fate diversity; instead, it increases the number of comparably sized basins.

This effect is accompanied by reduced dominance of any single fate ($p_{\max }=0.296$) and a lower Gini coefficient ($G=0.603$), indicating a flatter basin-size distribution and weaker fate-level structural canalization. Convergence times remain short, consistent with rapid commitment to terminal states, but these terminal states reflect defective meristem termination rather than normal reproductive differentiation. Quantitatively, *ag* produces a landscape that is diverse in basin structure yet biologically indeterminate in outcome.

*Summary of quantitative deformation modes.* Taken together, these metrics reveal that single homeotic mutations induce distinct quantitative signatures of landscape deformation. The *ap1* mutant reduces fate diversity and strongly increases dominance, the *pi* mutant redistributes basin mass without major changes in global structure, and the *ag* mutant increases effective fate number while flattening basin dominance. These patterns provide a quantitative complement to the attractor-level analysis and set the stage for examining non-additive effects in double mutants.

These quantitative differences are summarized in Table 3, which provides a compact comparison of dominance, diversity, and dynamical depth across genotypes.

**Table 3:** Global quantitative descriptors of epigenetic landscape structure under wild-type and single homeotic mutations. Metrics are computed over the full $2^{13}$-state space

Genotype	*H* (bits)	$N_{\textrm{eff}}$	$p_{\max }$	$p_{\textrm{top2}}$	*G*	Mean steps	Max steps
WT	2.129	4.38	0.374	0.737	0.655	4.11	9
*ap1*	1.468	2.77	0.469	0.927	0.651	3.39	7
*pi*	2.044	4.13	0.374	0.748	0.590	4.11	9
*ag*	2.283	4.87	0.296	0.589	0.603	3.16	7

To facilitate comparisons between landscapes arising from different genetic perturbations, we formalize indistinguishability as follows. Let $d(\mathcal {G}) \in \mathbb {R}^k$ denote the vector of global landscape descriptors reported in Tables 3 and 6 (e.g., *H*, $N_{\textrm{eff}}$, $p_{\max }$, $p_{\textrm{top2}}$, *G*, $\langle L \rangle$). Given a tolerance vector $\boldsymbol{\varepsilon } \in \mathbb {R}_{>0}^k$, we say that two landscapes $\mathcal {G}$ and $\mathcal {G}'$ are $\boldsymbol{\varepsilon }$-indistinguishable in descriptor space if

$$ |d_j(\mathcal {G}) - d_j(\mathcal {G}')| \le \varepsilon _j \quad \text {for all } j = 1, \ldots , k. $$

We use component-wise tolerances $\varepsilon _H = 10^{-3}$, $\varepsilon _N = 10^{-2}$, $\varepsilon _p = 10^{-3}$, and $\varepsilon _L = 10^{-2}$, with analogous tolerances for the remaining reported descriptors. For probability-based descriptors, basin fractions are quantized in multiples of $2^{-13}$, and the chosen tolerances are small relative to the separations observed among distinct mutant landscapes. Thus, indistinguishability is assessed at the level of the descriptor vector: two landscapes may be indistinguishable by these global summaries without being identical as state-transition graphs.

Table 4 resolves these quantitative patterns at the level of organ-identity classes. This per-organ view makes explicit how each single mutant redistributes basin mass within the floral sector. In the *ap1* mutant, sepal and petal attractor classes disappear in the model, with most basin mass assigned to the remaining inner-whorl identity classes. In the *pi* mutant, petal and stamen basins are eliminated and basin mass is redirected toward sepals and carpels: 20.12% of the state space converges to sepals and 74.80% to carpels, compared with 9.91% and 36.25% in wild type. In the *ag* mutant, stamen and carpel basins are eliminated and basin mass is concentrated on sepal- and petal-associated outcomes, including attractors with persistent *WUS* activity.

**Table 4:** Per-organ basin distribution under wild-type and single homeotic mutations. Percentages indicate the fraction of the full $2^{13}=8192$ state space converging to attractors of each identity class. *UFO*-on and *UFO*-off variants are aggregated within the corresponding organ class. For the *ag* mutant, sepal and petal totals include attractors with persistent *WUS* activity, reflecting defective meristem termination

Genotype	Attr.	Se.	Pe.	St.	Ca.	Infl.	*H*
WT	10	9.91	10.21	38.55	36.25	5.08	2.129
*ap1*	7	0.00	0.00	47.90	45.85	6.25	1.468
*pi*	8	20.12	0.00	0.00	74.80	5.08	2.044
*ag*	10	46.97	47.95	0.00	0.00	5.08	2.283

Se. = sepals; Pe. = petals; St. = stamens; Ca. = carpels; Infl. = inflorescence-associated attractors. All organ columns are percentages; *H* is in bits

### Double mutants and non-additive landscape deformation

Double homeotic mutants reveal strongly non-additive effects on the topology of the epigenetic landscape. Their landscapes are not simple superpositions of the corresponding single-mutant deformations; instead, they expose interactions between regulatory modules controlling fate accessibility, organ identity, and meristem termination. These interactions can collapse accessible identities onto restricted fate classes, preserve indeterminacy through persistent *WUS* activity, or redistribute basin mass across distinct terminal states. Such non-additive behavior is a hallmark of epistasis in genetic systems, where the phenotypic effect of a mutation depends on the presence or absence of other mutations (Phillips 2008; Lehner 2011).

To test whether the additional removal of *AP3* produces any global deformation beyond that induced by loss of *PI*, we performed a direct quantitative comparison between the *pi* single mutant and the *ap3–pi* double mutant.

**Table 5:** Direct quantitative comparison between the *pi* single mutant and the *ap3–pi* double mutant. Differences remain below numerical precision thresholds for all landscape descriptors

Metric	*pi*	*ap3–pi*	$|\Delta |$
# Attractors	8	8	0
Basin entropy *H* (bits)	2.044	2.044	$<10^{-3}$
$N_{\textrm{eff}}$	4.13	4.13	$<10^{-2}$
$p_{\max }$	0.374	0.374	$<10^{-3}$
$p_{\textrm{top2}}$	0.748	0.748	$<10^{-3}$
Mean steps $\langle L\rangle$	4.11	4.11	$<10^{-2}$
Max steps	9	9	0

As shown in Table 5, the *ap3–pi* double mutant is quantitatively indistinguishable from the *pi* single mutant at numerical precision. All global landscape descriptors coincide within the predefined tolerances; under the $\varepsilon$-indistinguishability criterion of Section 4.2, *ap3–pi* and *pi* are equivalent at the level of the Epigenetic Forest.

This equivalence follows from the AND-like structure of the B-function module in the Boolean rules (Appendix 1): B-target activation requires both *PI* and *AP3*. Once *PI*=0, B-function-dependent targets are already silenced, and an additional *AP3*=0 clamp does not further alter the global landscape descriptors. The resulting $\varepsilon$-indistinguishability is therefore a direct consequence of logical saturation in the B-function module. In the standard epistasis vocabulary, *PI* is epistatic to *AP3* with respect to global landscape structure (Phillips 2008; Lehner 2011). This behavior contrasts with double mutants involving *AG* or *AP1*, which affect distinct regulatory modules and produce non-additive deformations of basin topology and convergence dynamics.

In contrast, the *ag–pi* double mutant produces a qualitatively distinct deformation of the epigenetic landscape. All floral identities collapse onto sepal-like states, consistent with the combined loss of B- and C-function. This identity collapse is not accompanied by a reduction in basin entropy relative to the wild-type landscape (Table 6). Instead, the landscape partitions into large, symmetry-related basins corresponding to sepal identities with and without persistent *WUS* activity. As a result, substantial structural diversity is retained despite the drastic reduction in the repertoire of organ identities. This behavior reflects the persistence of *ag*-induced meristematic indeterminacy even in the complete absence of B-function.

The *ap1–ag* double mutant exhibits a distinct regime: it has the highest basin entropy among double mutants, while also showing the shortest convergence times (Table 6). In the terminology of Section 3.2.1, this combines strong temporal canalization with weak fate-level structural canalization: trajectories reach terminal states rapidly, but accessibility remains distributed across multiple large basins. Thus, the landscape is dynamically shallow without being fate-dominant.

Mechanistically, loss of *AP1* prevents early floral commitment, whereas loss of *AG* disrupts meristem termination. The resulting landscape retains both sepal- and petal-like terminal identities, each appearing in variants with repressed or persistent *WUS* expression. The corresponding Epigenetic Forest is shallow and rapidly convergent, yet divided into multiple large basins distinguished primarily by meristematic state rather than by organ identity.

Taken together, these results demonstrate that double mutants do not simply amplify the effects of single perturbations. Instead, they reveal distinct, non-additive modes of landscape deformation: saturation of the AND-like B-function module in *ap3–pi*, identity collapse with preserved *WUS*-dependent indeterminacy in *ag–pi*, and early commitment failure combined with defective termination in *ap1–ag*. In this sense, epistasis is expressed not only as a gene-level interaction, but also as a global topological property of the epigenetic landscape (Phillips 2008; Lehner 2011).

The Epigenetic Forest framework captures these regimes in a unified and quantitative manner (Table 6).

**Table 6:** Global epigenetic landscape metrics for double homeotic mutants of the floral GRN. Basin entropy is computed from the distribution of basin sizes over the full $2^{13}$ state space. “Largest” is the size (in states) of the largest basin; Mean and Max are mean and maximum convergence steps. In *ap1–ag*, petal-like identities appear because loss of *AP1* and *AG* leaves B-function (*PI*, *AP3*) active in some states; the dominant basins are sepal- and petal-like, with or without persistent *WUS*

Genotype	# Attr.	*H* (bits)	Dominant identities	Largest	Mean	Max
AP3–PI	8	2.044	Sepals / Carpels	3064	4.11	9
AG–PI	8	2.244	Sepals ± WUS	2424	3.15	7
AP1–AG	8	2.292	Sepals, Petals ± WUS	2400	2.93	6

### Attractor identities and gene expression consistency

The biological interpretation of the Epigenetic Forest depends on whether the attractors recovered under single and double perturbations correspond to known expression programs. In floral development, this comparison is especially direct because organ identities are defined by well-characterized combinations of transcription factor activity. We therefore examine whether the attractors recovered under homeotic perturbations reproduce known organ-specific expression signatures.

Genome-wide expression analyses by Wellmer and Riechmann (2004) provide a canonical reference for this validation. Their study demonstrated that floral organs in *Arabidopsis thaliana* are associated with distinct transcriptional programs, with sepals and petals exhibiting relatively modest specialization, and stamens and carpels displaying highly specific expression profiles. Organs arising in homeotic mutants were shown to largely recapitulate the gene expression signatures of their wild-type counterparts, rather than exhibiting intermediate or mixed transcriptional states.

The Epigenetic Forest framework reproduces this qualitative pattern. In the *pi* mutant, the surviving sepal and carpel attractors match the wild-type expression patterns of these organs; no attractors with partial or hybrid B-function signatures are observed (Section 4.2). In the *ag* mutant, the loss of AG-mediated repression of *WUS* (Lenhard and Laux 2001; Laux et al. 1996) is captured by attractors that differ from wild-type sepal or petal states exclusively by activation of the *WUS* node, with all organ-identity genes retaining their canonical configurations.

These attractors therefore represent minimal deviations from wild-type organ identities, characterized by a single-bit difference corresponding to persistent *WUS* expression. In the *ag* landscape, clamping *AG*=0 modifies the update map and replaces the affected wild-type fixed points with nearby fixed points that retain the same organ-identity readout but differ in their meristematic component. Thus, what is preserved is the regulatory configuration of organ-identity genes, corresponding to canonical A/B/C patterns, whereas the original wild-type attractors themselves are not fixed points of the mutant dynamics.

We refer to this pattern as identity-gene preservation under attractor relocation. The meristem-termination program fails because trajectories that would normally end in *WUS*=0 reproductive attractors are redirected toward terminal states with persistent *WUS* activity. This combination of preserved organ-identity readout and altered meristematic state can be interpreted as a breakdown of homeorhetic convergence: trajectories no longer reach the wild-type termination state, but converge instead to nearby *WUS*=1 attractors that retain organ-identity structure together with meristematic activity.

**Table 7:** Qualitative comparison of attractor emergence and accessibility under single homeotic mutations

Mutant	New attractors	Attractors lost	Landscape deformation mode
*ap1*	No	Floral organ identities	Restriction of accessibility: trajectories are redirected toward pre-existing inflorescence states without emergence of new attractors.
*pi*	Yes	Petals, stamens	Identity collapse: B-function–dependent fates eliminated and redistributed among sepal- and carpel-like basins.
*ag*	Yes	Reproductive identities	Termination failure: emergence of perianth-like attractors with persistent *WUS* activity and sustained indeterminacy.

Taken together, these results show that the Epigenetic Forest preserves the core gene expression logic of floral organ identity under homeotic perturbations (Table 7). The recovered attractors correspond either to canonical wild-type expression programs or to experimentally documented variants arising from specific regulatory failures. This consistency supports their interpretation as biologically meaningful cell-fate representations and motivates the use of the Epigenetic Forest as a discrete analogue of the floral epigenetic landscape.

### Landscape divergence from wild type

While entropy- and basin-based metrics characterize internal properties of each epigenetic landscape, they do not directly quantify how far a mutant landscape departs from the wild-type organization as a whole. To address this question, we compare landscapes as probability distributions over coarse-grained identity classes using the Jensen–Shannon (JS) divergence.

The JS divergence provides a symmetric, bounded measure of dissimilarity between two probability distributions and is therefore well suited for comparing epigenetic landscapes at the global level. In this context, each landscape is represented by the basin-weighted probability vector over organ identity classes (sepals, petals, stamens, carpels, and inflorescence states). A value of $\textrm{JS}=0$ indicates identical accessibility of fates, whereas larger values indicate increasing redistribution of basin mass relative to the wild type.

Table 8 reports the JS divergence between the wild-type landscape and each mutant condition.

**Table 8:** Jensen–Shannon divergence (in bits) between wild-type and mutant epigenetic landscapes. Distributions are computed from basin-weighted probabilities over coarse-grained identity classes

Genotype	$\textrm{JS}(P_{\textrm{WT}}, P_{\textrm{mut}})$
*ap1*	0.188
*pi*	0.144
*ag*	0.103
*ap3–pi*	0.144
*ag–pi*	0.214
*ap1–ag*	0.226

Several features are immediately apparent. Among single mutants, the *ap1* perturbation produces the largest divergence from the wild-type landscape, reflecting the global redirection of trajectories toward inflorescence states and the loss of access to floral identities. In contrast, the *ag* mutant shows the smallest divergence despite its pronounced developmental phenotype, consistent with the fact that most basin mass remains concentrated in perianth-associated identities, albeit with altered meristematic status.

The *pi* mutant occupies an intermediate position: although B-function identities are eliminated, the overall redistribution of basin mass remains closer to wild type than in *ap1*, reflecting a collapse of identity repertoire without drastic reweighting of floral versus inflorescence domains.

Double mutants exhibit systematically larger JS divergences than their single-mutant counterparts, highlighting the non-additive nature of landscape deformation. In particular, the *ag–pi* and *ap1–ag* mutants display the strongest divergence from wild type, corresponding to landscapes in which identity collapse, altered commitment timing, and defective termination act simultaneously to redistribute basin accessibility.

Taken together, the JS divergence provides a compact, landscape-level summary that complements basin entropy and dominance metrics. Whereas entropy and Gini coefficients characterize internal structure and canalization, the JS divergence directly quantifies how far a mutant landscape departs from the wild-type developmental organization. In this sense, it closes the quantitative analysis by placing all perturbations on a common, global scale of epigenetic deformation.

### Global comparison of epigenetic landscape deformation

**Fig. 11:**
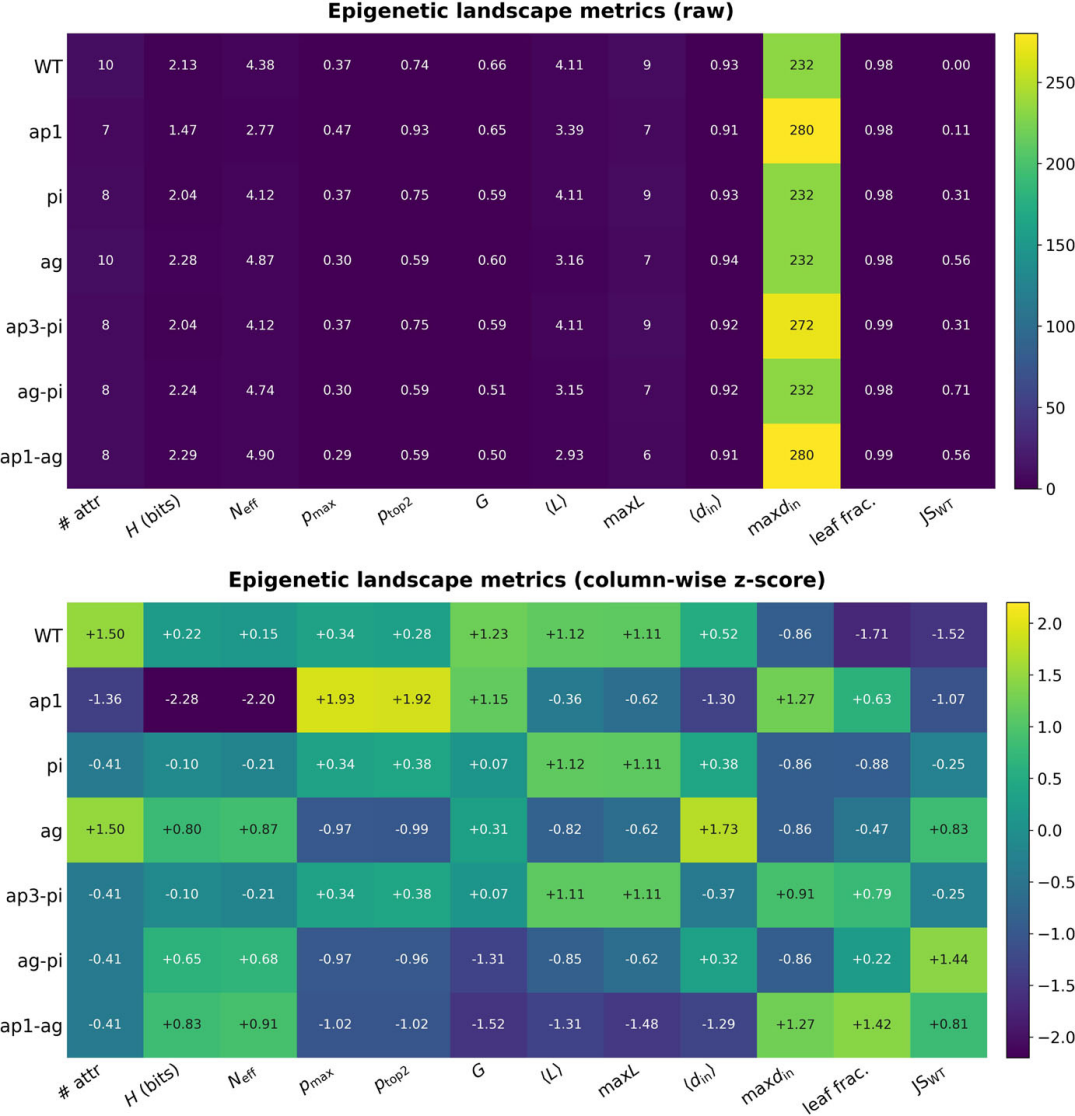
Global comparison of epigenetic landscape deformation across genotypes. (**a**) Heatmap of raw metric values. (**b**) Column-wise *z*-score normalization highlighting relative deviations from the mean for each metric. Rows correspond to genotypes (wild type, single mutants, and double mutants), and columns to quantitative descriptors of landscape structure

To synthesize the multiple quantitative descriptors introduced above, we performed a global comparison of wild-type, single, and double mutant epigenetic landscapes using a unified metric heatmap (Figure 11).

The heatmap reveals structured patterns across both genotypes and metrics. Single mutants and double mutants occupy distinct regions of the metric space, with double mutants exhibiting the strongest deviations from wild type across multiple, complementary descriptors. Notably, no single metric alone captures all aspects of landscape deformation, underscoring the need for a multidimensional characterization of epigenetic landscapes.

## Discussion

The Epigenetic Forest framework provides a discrete and exhaustive representation of the global dynamics of gene regulatory networks by organizing the full state transition graph into basins of attraction associated with stable gene expression patterns. In previous work, this representation was introduced as a combinatorial analogue of Waddington’s epigenetic landscape (Waddington 2014; Huang 2009, 2012), offering an exhaustive description of fate accessibility and differentiation trajectories induced by regulatory logic.

In the present study, we build upon this framework to analyze how homeotic perturbations reshape the structure of the epigenetic landscape. By focusing on single and double homeotic mutants of the *Arabidopsis thaliana* floral GRN, we move beyond the identification of terminal fates and instead characterize how genetic perturbations deform the landscape at a structural level. In particular, we examine how mutations affect attractor accessibility, basin organization, convergence depth, dominance relations, and global fate distributions within the Epigenetic Forest.

This analysis shows that homeotic genes contribute to development both through local identity specification and through their effects on the global organization of the epigenetic landscape. Different genes induce qualitatively distinct modes of landscape deformation, including restricted fate accessibility without emergence of new attractors, loss of B-function-dependent identity classes, appearance of additional stable states associated with defective termination, and non-additive epistatic effects in double mutants. These deformation modes provide a structural interpretation of developmental robustness, access to alternative outcomes, and regulatory failure that is less visible from local rule inspection or attractor identity alone.

### Structural interpretation of homeotic mutants

Our results show that single homeotic mutations deform the Epigenetic Forest through distinct structural mechanisms. Loss of *AP1* primarily restricts fate accessibility: floral attractor classes are lost without the emergence of new terminal identities, and trajectories are shifted toward the inflorescence-associated region of state space. Loss of *PI* removes B-function-dependent attractors and redistributes basin mass among the remaining A-only and C-only identity classes, with no emergence of intermediate B-function states. Loss of *AG* produces a different deformation: organ-identity configurations are partly retained, but terminal states acquire persistent *WUS* activity, providing a landscape-level signature of defective meristem termination and disrupted homeorhetic convergence.

Thus, the three single mutants affect different structural features of the landscape: access to floral fates in *ap1*, availability of B-function identities in *pi*, and completion of meristem termination in *ag*. In this sense, the Epigenetic Forest separates three regulatory roles that would be less visible from attractor labels alone: commitment, identity specification, and termination.

### Non-additivity and epistasis in double mutants

Double mutants reveal that landscape deformation is strongly non-additive. The *ap3–pi* double mutant is $\varepsilon$-indistinguishable from the *pi* single mutant in descriptor space, showing that once *PI*=0, additional removal of *AP3* does not further alter the global landscape descriptors. This reflects logical saturation of the B-function module and provides a landscape-level interpretation of epistasis that goes beyond gene-by-gene interaction diagrams. This use of saturation is distinct from classical functional redundancy: it does not imply mutual buffering between *PI* and *AP3*, but rather that the AND-like B-function rule is already inactive once *PI*=0.

By contrast, the *ag–pi* double mutant collapses floral identity onto sepal-like states while preserving substantial structural diversity through persistent *WUS*-dependent indeterminacy. Similarly, the *ap1–ag* mutant combines failure of early floral commitment with defective termination, yielding a landscape that is shallow in convergence depth yet strongly partitioned in state space. Together, these double mutants show that regulatory perturbations affecting floral commitment, B-function identity specification, and meristem termination leave distinguishable signatures in the descriptor space of the Epigenetic Forest. Access to floral fates reflects the contribution of *AP1*; the presence or absence of B-function-dependent attractors reflects the contribution of *PI*; and the absence or persistence of *WUS*=1 attractors reflects *AG*–SEP-mediated termination. In single mutants, these signatures appear individually, whereas in double mutants they combine into composite, non-additive patterns. Thus, the double mutants act as diagnostic perturbation combinations rather than simple stronger perturbations: each genotype preserves enough structure to identify which regulatory axis has been removed and which remains active. In particular, *ag–pi* retains the *ag*-associated *WUS* signature together with the *pi*-associated loss of B-function attractors, while *ap1–ag* combines the loss of floral basins induced by *ap1* with the defective-termination signature associated with *ag*.

### Quantitative signatures of fate concentration, fate diversity, and dynamical depth

The quantitative analysis introduced here compares landscape deformation along the operational axes defined in Section 3.2.1. Basin entropy and the effective number of fates quantify fate diversity; $p_{\max }$, $p_{\textrm{top2}}$, and the Gini coefficient quantify fate-level structural canalization, understood as the concentration of state-space accessibility into a few dominant basins; convergence statistics quantify temporal canalization, namely the speed of commitment toward terminal fates; and Jensen–Shannon divergence measures the global departure of each mutant fate distribution from the wild-type reference.

Together, these descriptors define complementary axes of landscape organization. They distinguish whether a perturbation reduces the number of accessible fates, concentrates accessibility into dominant basins, accelerates or delays convergence, or redistributes state-space probability across fate classes.

The heatmap-based synthesis of these metrics highlights how single and double mutants cluster into distinct deformation regimes, making explicit the structural consequences of regulatory perturbations at the level of the full state space.

### Modeling assumptions and robustness

Our analysis is based on a deterministic Boolean representation of the floral GRN with synchronous updates. This choice enables exhaustive exploration of the full $2^{13}$-state space and yields a unique deterministic transition digraph, allowing an exact basin decomposition. The rooted-tree (“forest”) structure reported here is therefore a property of the synchronous transition digraph induced by *F*. We use the Boolean rules of Espinosa-Soto et al. (2004) (2004) as a previously validated Boolean model of this network; extensions to more recent or extended GRN versions, including ABCDE-aware models that incorporate SEPALLATA E-function genes (Pelaz et al. 2000; Theißen and Saedler 2001; Causier et al. 2010; Stefan De Folter et al. 2005) and updated regulatory interaction sets such as those compiled in subsequent analyses (Sánchez-Corrales et al. 2010; Alvarez-Buylla et al. 2010), could be explored in future work.

Fixed-point states remain fixed under any partial-update scheme consistent with the same local Boolean rules, since they satisfy $f_i(x)=x_i$ for all *i*. Basin proportions and trajectory-depth statistics, however, depend on the update scheme: under fully asynchronous or stochastic dynamics, the system is no longer described by a single-valued map $F:\mathcal {S}\rightarrow \mathcal {S}$, but by a relation or stochastic process (Klemm and Bornholdt 2005; Shmulevich et al. 2002). The robustness and epistasis results reported here therefore concern regulatory-logic constraints, attractor identities, and basin organization within the synchronous reference framework. Extensions to asynchronous or stochastic update schemes provide a natural continuation of the present analysis.

The synchronous framework provides a reference description of landscape structure rather than a detailed temporal model of floral development. By making the state transition graph finite and single-valued, it allows fate accessibility, basin organization, and convergence depth to be computed exhaustively. In this respect, the Epigenetic Forest complements continuous, stochastic, and energy-based landscape models.

### Relation to other landscape-based approaches

Epigenetic landscapes have been explored using a wide range of mathematical formalisms, including continuous dynamical systems, stochastic differential equations, and potential-like constructions derived from probabilistic dynamics. These approaches have yielded important insights into local stability, noise-induced transitions, and barrier heights between fates (Huang 2012; Ferrell 2012). More recent work has revisited Waddington’s landscape from a dynamical-systems perspective and from data, including the data-driven reconstruction of developmental landscapes from single-cell measurements (Cislo et al. 2025), which provides an explicit dynamical-systems counterpart to the discrete construction proposed here.

The Epigenetic Forest offers a complementary, combinatorial perspective. Instead of inferring an underlying potential, it explicitly enumerates all admissible trajectories and their convergence to terminal states. In this framework, the epigenetic landscape is represented as a finite topological object determined by the regulatory logic of the network.

This makes fate accessibility, basin dominance, canalization, and epistasis directly computable and interpretable at a global level.

By focusing on how homeotic perturbations reshape the forest structure, the present work highlights aspects of developmental organization–such as non-additive epistasis and identity pruning–that are difficult to extract from local or energy-based descriptions alone. The Epigenetic Forest framework thus offers a concrete way to analyze how regulatory logic constrains development at the level of the full state space and how genetic perturbations reorganize it in biologically interpretable ways.

### Evolutionary implications of epigenetic forest deformation

From an evolutionary perspective, the deformation modes identified in the Epigenetic Forest suggest that robustness to mutation and access to alternative developmental outcomes are encoded in the global structure of regulatory logic, not only in the local function of individual genes. Homeotic mutations do not merely alter terminal identities; they reshape fate accessibility, basin dominance, and convergence structure across the entire state space.

Landscapes dominated by a small number of large basins correspond to highly canalized developmental programs, which are expected to be robust under perturbation. Conversely, landscapes with higher basin entropy or persistent indeterminacy increase the accessibility of alternative developmental outcomes, potentially enabling exploratory phenotypes at the cost of developmental precision. Selection can therefore act not only on specific attractors, but on the global topology of the epigenetic forest itself.

Within this framework, homeotic genes can be interpreted as regulatory control points that stabilize, restrict, or redirect large regions of state space. The loss of B-function identities in *pi* and *ap3–pi*, together with the emergence of indeterminate *WUS*-positive states in *ag*, illustrates how localized regulatory perturbations can induce large-scale changes in landscape topology. Such sensitivity suggests a mechanism by which regulatory changes may contribute both to evolutionary innovation and to developmental constraint, through changes in fate accessibility, basin dominance, and terminal-state organization rather than through gradual modulation of individual expression levels alone.

### Experimental predictions

Because the Epigenetic Forest exhaustively characterizes fate accessibility, dominance, and basin organization within the Boolean model, it yields predictions that can be tested against gene expression distributions and mutant developmental outcomes. These predictions concern global structural properties of the landscape rather than fine-grained temporal dynamics along individual trajectories.

First, the framework predicts that double mutants combining early commitment defects with impaired termination should exhibit increased structural fate diversity without access to genuinely novel identity classes. In particular, the *ap1–ag* mutant is expected to display a heterogeneous population of perianth-like and inflorescence-like transcriptional states, reflecting the coexistence of failed floral commitment and sustained meristematic activity. This prediction can be tested by single-cell or bulk transcriptomic profiling of late floral tissues, which should reveal increased expression variability across canonical outer-organ programs without evidence of hybrid or intermediate identities.

Second, the model predicts that partial perturbations of termination regulators should induce graded and predictable changes in landscape structure. For example, a partial reduction of *WUS* activity in the *ag* background is expected to decrease basin entropy and restore dominance relations toward a subset of perianth-associated basins, effectively shifting the mutant landscape toward a more canalized regime. Experimentally, this would manifest as reduced transcriptional heterogeneity and diminished persistence of meristematic markers in inner floral whorls, without altering the set of accessible identity classes.

Together, these predictions illustrate how the Epigenetic Forest framework enables a direct translation from regulatory logic to experimentally observable constraints on developmental outcomes. By linking genetic perturbations to global changes in landscape topology, the model provides a principled basis for designing experiments that probe not only which fates arise, but how developmental constraints themselves are reorganized at the level of the full state space.

### Comparison with other discrete gene regulatory network models

Discrete gene regulatory network models have been extensively used to study floral development, notably in the Boolean frameworks developed by Mendoza, Álvarez-Buylla, and collaborators, beginning with the seminal Arabidopsis floral GRN model (Sánchez-Corrales et al. 2010) and subsequent extensions and analyses (Espinosa-Soto et al. 2004; Álvarez-Buylla et al. 2008). These models successfully reproduce experimentally observed attractors and provide valuable insights into local stability, robustness, and gene-level regulatory interactions underlying floral organ specification.

There is also a substantial body of more general work on Boolean network state graphs and basins of attraction, going back to Kauffman’s foundational analysis of random Boolean networks (Kauffman 1969). Albert and Othmer (2003) used basin structure to analyze the segment-polarity gene network in *Drosophila* (Xiao and Dougherty 2007) systematically studied how function (rule) perturbations reshape Boolean state-transition graphs, with explicit attention to attractor reorganization (Wang et al. 2012) reviewed basin- and attractor-based analyses of Boolean models in systems biology (Joo et al. 2018) developed Hamming-neighbor measures of dynamic stability of cell states in Boolean networks (Klarner et al. 2020) introduced computational tools for basins of attraction and commitment sets in asynchronous Boolean networks. The Epigenetic Forest framework lies in this lineage but shifts the focus from individual attractors to the global organization of the full state transition graph, in two specific respects. First, our perturbations are biologically grounded loss-of-function clamps in a well-characterized floral GRN, and each landscape deformation is connected to a documented mutant phenotype. Second, we organize the comparison along the multidimensional descriptor space introduced above and use it to formalize an explicit notion of $\varepsilon$-indistinguishability that makes epistasis directly visible at the level of the global landscape. Whereas previous Boolean GRN models typically emphasize the existence, stability, and biological interpretation of specific fates, the Epigenetic Forest characterizes how the entire state space is partitioned into basins, how dominant fates emerge, and how genetic perturbations reshape accessibility, dominance, and canalization at a system-wide level.

This distinction is particularly relevant for the analysis of epistasis and non-additivity in double mutants. The Epigenetic Forest represents genetic interactions as global changes in landscape organization, including descriptor-level equivalence between *ap3–pi* and *pi*, loss of B-function-dependent identity classes, redistribution of basin mass, and altered convergence structure. In this sense, the present framework is not an alternative to Boolean GRN models, but an analytical layer built on top of them: it uses the same regulatory logic to reveal structural properties of developmental landscapes that are not captured by local rule inspection or by attractor identity alone.

## Conclusions

In this work, we analyzed how homeotic mutations reshape the developmental landscape of *Arabidopsis thaliana* through the full state-transition structure of its Boolean floral GRN. Building on the Epigenetic Forest framework, we quantified how single and double perturbations deform basin structure, fate accessibility, and convergence. The comparison was based on complementary descriptors of basin organization, fate diversity, dominance, convergence, and divergence from wild type.

Our results show that distinct regulatory genes induce qualitatively different deformation modes. Loss of *AP1* restricts access to floral fates without reconfiguring the attractor set; loss of *PI* collapses floral identity by eliminating B-function–dependent fates; loss of *AG* generates additional stable states associated with defective meristem termination via persistent *WUS* activity. These regimes are reflected in attractor composition, basin dominance, effective fate number, and convergence depth. Double mutants exhibit strongly non-additive, epistatic effects: *ap3–pi* is $\varepsilon$-indistinguishable from *pi* in descriptor space, while *ag–pi* and *ap1–ag* diverge sharply from wild type. The metrics and comparisons presented in the Results and Discussion sections make these deformation modes explicit and comparable across genotypes.

Within the Epigenetic Forest framework, the epigenetic landscape is represented as a finite, computable object whose topology is determined by the specified regulatory logic. Fate accessibility, canalization, dominance, and epistasis can therefore be quantified as features of the global state transition graph. Developmental trajectories are constrained by this topology, so robustness and failure can be characterized at the level of forest organization rather than only through local regulatory interactions. Although illustrated here for floral development, the approach applies to any Boolean network with finite deterministic dynamics and provides a general way to analyze how genetic perturbations reshape developmental landscapes.

## Appendix A Boolean Update Rules

We list the complete set of Boolean update functions defining the GRN dynamics. All nodes are updated synchronously under deterministic Boolean dynamics. Logical operators $\wedge$, $\vee$, and $\lnot$ denote AND, OR, and NOT, respectively. Boolean update rules correspond to minimal logical expressions derived from experimentally constrained truth tables.

All variables take values in $\{0,1\}$ and each rule specifies the next state at time $t{+}1$ as a function of the current state at time *t*. We treat *UFO* as a constitutively active input (i.e., $UFO(t)=1$ for all *t*).

The Boolean update rules are written in a minimized logical form that is exactly equivalent to the complete experimentally constrained truth tables used in previous validated models.

$$\begin{aligned} FUL(t{+}1)&= \lnot AP1(t)\wedge \lnot TFL1(t), \\ FT(t{+}1)&= \lnot EMF1(t), \\ AP1(t{+}1)&= \lnot AG(t)\wedge \Big (\lnot TFL1(t) \vee (FT(t)\wedge LFY(t)) \\&\quad \vee (FT(t)\wedge \lnot PI(t)) \vee (FT(t)\wedge \lnot AP3(t)) \\&\hspace{22mm}\vee (LFY(t)\wedge \lnot PI(t)) \vee (LFY(t)\wedge \lnot AP3(t))\Big ), \\ EMF1(t{+}1)&= \lnot LFY(t), \\ LFY(t{+}1)&= \lnot EMF1(t)\vee \lnot TFL1(t), \\ AP2(t{+}1)&= \lnot TFL1(t), \\ WUS(t{+}1)&= WUS(t)\wedge (\lnot AG(t)\vee \lnot SEP(t)), \\ AG(t{+}1)&= \lnot AP2(t)\wedge \lnot EMF1(t)\wedge (AG(t)\vee LFY(t)\vee WUS(t)) \\&\quad \wedge (AG(t)\vee LFY(t)\vee \lnot SEP(t)), \\ SEP(t{+}1)&= LFY(t), \\ PI(t{+}1)&= (LFY(t)\wedge (AG(t)\vee AP3(t))) \\&\quad \vee (AP3(t)\wedge PI(t)\wedge SEP(t)\wedge (AG(t)\vee AP1(t))), \\ AP3(t{+}1)&= (LFY(t)\wedge UFO(t)) \\&\quad \vee (AP3(t)\wedge PI(t)\wedge SEP(t)\wedge (AG(t)\vee AP1(t))), \\ TFL1(t{+}1)&= EMF1(t)\wedge \lnot LFY(t)\wedge \lnot AP2(t) \\&\quad \wedge \lnot AP1(t), \\ UFO(t{+}1)&= 1. \end{aligned}$$

## Appendix B Algorithmic construction of Epigenetic Forest metrics

This appendix provides a compact implementation-oriented reference for how the Epigenetic Forest and the global landscape metrics reported in the main text were computed. The goal is not to present exhaustive software, but to document the computational pipeline in a transparent, reproducible manner.

The procedure below corresponds to the exhaustive enumeration algorithm used in our previous implementation, rewritten here in a model-independent and formal way.

### B.1 State space, update map, and clamping

We consider the finite state space $\mathcal {S} = \{0,1\}^{13}$ and the synchronous Boolean update map $F: \mathcal {S} \rightarrow \mathcal {S}$. Each state $x \in \mathcal {S}$ has a unique successor *F*(*x*), which defines a functional state transition graph. Loss-of-function mutants are implemented by clamping selected genes to 0 at every step (output-only clamping): for a set of clamped coordinates $C \subset \{1,\ldots ,13\}$, the mutant dynamics are given by $\widetilde{F}^{(C)}(x) = \textrm{clamp}_{C}(F(x))$, where $(\textrm{clamp}_{C}(y))_i = 0$ if $i \in C$ and $(\textrm{clamp}_{C}(y))_i = y_i$ if $i \notin C$. All computations are performed by exhaustive enumeration over all $2^{13}$ states.

### B.2 Forest decomposition and basin assignment

For each initial condition $x \in \mathcal {S}$, the trajectory $x, \widetilde{F}^{(C)}(x), (\widetilde{F}^{(C)})^2(x), \ldots$ is iterated until it reaches a fixed point *a* satisfying $\widetilde{F}^{(C)}(a) = a$. Under the dynamics considered here, all attractors are fixed points (no cycles of length $> 1$ occur). Each fixed point *a* defines a basin of attraction $\mathcal {B}(a) = \{ x \in \mathcal {S}: (\widetilde{F}^{(C)})^t(x) = a \text { for some } t \ge 0 \}$, and the Epigenetic Forest is the collection of rooted directed trees induced by these basins.

### B.3 Global metrics

Let $\mathcal {A}$ be the set of fixed-point attractors, $m = |\mathcal {A}|$, and $p_a = |\mathcal {B}(a)|/2^{13}$ the basin fraction of attractor *a* (so that $\sum _{a \in \mathcal {A}} p_a = 1$). We report the following global descriptors:

- **Basin entropy** (bits): $H = -\sum _{a \in \mathcal {A}} p_a \log _2 p_a$.
- **Effective number of fates**: $N_{\textrm{eff}} = 2^H$.
- **Dominance**: $p_{\max } = \max _{a \in \mathcal {A}} p_a$ and $p_{\textrm{top2}} = p_{(1)} + p_{(2)}$, where $p_{(1)} \ge p_{(2)} \ge \cdots$ are the ordered basin fractions.
- **Inequality** (Gini coefficient): $G = \frac{1}{2m} \sum _{i=1}^{m} \sum _{j=1}^{m} |p_i - p_j|$.
- **Convergence depth**: for each $x \in \mathcal {S}$, let *L*(*x*) be the number of synchronous updates needed to reach its terminal fixed point; we report $\langle L \rangle = \frac{1}{2^{13}} \sum _x L(x)$ and $\max _x L(x)$.
- **Landscape-level divergence**: Jensen–Shannon divergence between wild-type and mutant coarse-grained fate distributions (grouped by organ/meristem classes), $\textrm{JS}(P,Q) = \frac{1}{2} D_{\textrm{KL}}(P \Vert M) + \frac{1}{2} D_{\textrm{KL}}(Q \Vert M)$ with $M = \frac{1}{2}(P+Q)$, using $\log _2$ so that JS is reported in bits.

Numerical indistinguishability refers to agreement of the reported global descriptors within the prescribed tolerances (Section 4.2). This descriptor-level comparison does not require equality of the underlying state transition graphs.

### B.4 Reference pseudocode (single and double mutants)

The following procedure summarizes the exhaustive computation for any genotype defined by a clamp set *C*.

**Input:** clamp set *C* (genes fixed to 0); optional: wild-type coarse fate distribution $P_{\textrm{WT}}$ (for JS).

**Steps:** (1) Build the successor map $\textrm{next}[x] = \widetilde{F}^{(C)}(x)$ for all $x \in \mathcal {S}$. (2) Identify fixed points $\mathcal {A} = \{ a: \textrm{next}[a] = a \}$. (3) For each state *x*, iterate $x \rightarrow \textrm{next}[x] \rightarrow \cdots$ until reaching some $a \in \mathcal {A}$; record $\textrm{attr}[x] = a$ and $L[x] = \text {number of steps}$. (4) Compute basin sizes $\textrm{size}[a] = \#\{ x: \textrm{attr}[x] = a \}$ and fractions $p_a = \textrm{size}[a]/2^{13}$. (5) Compute *H*, $N_{\textrm{eff}}$, $p_{\max }$, $p_{\textrm{top2}}$, *G*, $\langle L \rangle$, $\max L$. (6) Group basins by organ/meristem identity to obtain the coarse fate distribution $P_C$. (7) If $P_{\textrm{WT}}$ is provided, compute $\textrm{JS}(P_{\textrm{WT}}, P_C)$.

**Output:** attractors $\mathcal {A}$, basin sizes, and global metrics.
